# Structural alteration of DNA induced by viral protein R of HIV-1 triggers the DNA damage response

**DOI:** 10.1186/s12977-018-0391-8

**Published:** 2018-01-16

**Authors:** Kenta Iijima, Junya Kobayashi, Yukihito Ishizaka

**Affiliations:** 1 Department of Intractable Diseases, National Center for Global Health and Medicine, 1-21-1 Toyama, Shinjuku-ku, Tokyo, 162-8655 Japan; 2 Department of Genome Repair Dynamics, Radiation Biology Center, Kyoto University, Yoshidakonoe-cho, Sakyo-ku, Kyoto, 606-8501 Japan

## Abstract

**Background:**

Viral protein R (Vpr) is an accessory protein of HIV-1, which is potentially involved in the infection of macrophages and the induction of the ataxia-telangiectasia and Rad3-related protein (ATR)-mediated DNA damage response (DDR). It was recently proposed that the SLX4 complex of structure-specific endonuclease is involved in Vpr-induced DDR, which implies that aberrant DNA structures are responsible for this phenomenon. However, the mechanism by which Vpr alters the DNA structures remains unclear.

**Results:**

We found that Vpr unwinds double-stranded DNA (dsDNA) and invokes the loading of RPA70, which is a single-stranded DNA-binding subunit of RPA that activates the ATR-dependent DDR. We demonstrated that Vpr influenced RPA70 to accumulate in the corresponding region utilizing the LacO/LacR system, in which Vpr can be tethered to the LacO locus. Interestingly, RPA70 recruitment required chromatin remodelling via Vpr-mediated ubiquitination of histone H2B. On the contrary, Q65R mutant of Vpr, which lacks ubiquitination activity, was deficient in both chromatin remodelling and RPA70 loading on to the chromatin. Moreover, Vpr-induced unwinding of dsDNA coincidently resulted in the accumulation of negatively supercoiled DNA and covalent complexes of topoisomerase 1 and DNA, which caused DNA double-strand breaks (DSBs) and DSB-directed integration of proviral DNA. Lastly, we noted the dependence of Vpr-promoted HIV-1 infection in resting macrophages on topoisomerase 1.

**Conclusions:**

The findings of this study indicate that Vpr-induced structural alteration of DNA is a primary event that triggers both DDR and DSB, which ultimately contributes to HIV-1 infection.

**Electronic supplementary material:**

The online version of this article (10.1186/s12977-018-0391-8) contains supplementary material, which is available to authorized users.

## Background

Combined antiretroviral therapy (cART) for human immunodeficiency virus-1 (HIV-1)-positive patients suppresses viral replication to a non-detectable level, thereby preventing immunodeficiency caused by T cell depletion. Unfortunately, the interruption of the cART regimen allows the expansion of viral replication from long-lived reservoir cells [1]. Because macrophages comprise the major cell population involved in the formation of viral reservoirs [2, 3], understanding the mode of viral infection in resting macrophages is crucial.

*Viral protein R* (*Vpr*) is an accessory gene of HIV-1, which encodes a virion-associated nuclear protein that is made up of 96 amino acids (aa) [4] and can facilitate viral infection in resting macrophages [5–8]. The induction of cell-cycle abnormality at the G_2_/M phase is a well-investigated function of Vpr among its pleiotropic activities [9]. Notably, Vpr induces a DNA damage response (DDR) involving ATR/ATRIP-Chk1 activation, the phosphorylation of histone H2AX (γH2AX) and the formation of BRCA1, 53BP1, RAD51 and FANCD2 foci [10–14]. Structural analyses revealed that Vpr contains three alpha helices with self-dimerisation properties along with a flexible carboxy (C)-terminal region with a basic stretch that is involved in DNA binding [15, 16], both of which were attributable to the cell-cycle abnormalities at the G_2_/M phase [17]. Originally, Vpr-induced G_2_/M arrest was proposed to contribute to viral infection by delaying cell death, thereby providing longer periods for viral replication [18]; however, its biological significance in resting macrophages remains largely unclear.

Regarding the upstream events that potentially trigger Vpr-induced DDR, several lines of evidence suggest that Vpr expression provokes replication stress, thereby inducing chromatin loading of the replication protein A 70-kDa subunit (RPA70), which is a DNA-binding subunit of the single-strand DNA (ssDNA) binding heterotrimeric protein [12, 19]). Considering that DNA binding of RPA70 triggers the ATR/ATRIP-Chk1 pathway responsible for G_2_/M cell-cycle arrest [20], it is imperative to determine the mechanism of RPA70 loading onto the chromatin. Furthermore, cellular ubiquitination by Vpr is also required for DDR activation: the Q65R mutant of Vpr, which cannot bind DDB1/VprBP, an adaptor protein for Cul4 E3-ligase, is defective for the G_2_/M checkpoint activation and cellular ubiquitination [21–24]. Although these findings imply that Vpr modulates RPA70 loading onto the chromatin in close functional association with the DDB1/VprBP-Cul4-dependent ubiquitination pathway, no cellular targets of Vpr-dependent ubiquitination have yet been identified.

For determining whether Vpr influences the structural alteration of DNA, Kichler et al. [25] performed an electron microscopic study and proposed that the C-terminal moiety of Vpr can aggregate plasmid DNA. Moreover, Lyonnais et al. [26] found that Vpr mediates the bridging and stretching of DNA helices. These observations strongly suggest that Vpr is capable of altering DNA structures. Generally, aberrant DNA structures interfere with DNA replication and transcription and may cause DNA damage. Such molecular consequences have been well-characterised in the context of DNA topological stress induced by camptothecin (CPT) and etoposide [27], which inhibit topoisomerase 1 (Topo1) and Topo2, respectively. Topo1 is involved in the relaxation of excess supercoils on dsDNA, whereas Topo2 is involved in the disentangling of dsDNA strands through cycles of cleavage and re-ligation. Chemical inhibition of re-ligation after relief of DNA distortions results in the formation of covalent complexes between Topo1 or Topo2 and cleaved DNA ends, which potentially induce DNA double-strand breaks (DSBs) due to subsequent collision of replication or transcription [27, 28].

Because Vpr itself does not possess DNA cleavage activity, Vpr must induce the DDR with the aid of host factor(s) [16]. Several cellular proteins, including UNG2, HTLF and SLX4 have been suggested as candidates for participation in Vpr-induced DDR [14, 29, 30]. UNG2 and HTLF are DNA repair proteins, and suppression of their functions leads to aggravation of DNA damage [29, 30]. On the other hand, Laguette et al. [14] suggested that Vpr causes the premature activation of the SLX4-Mus81/Eme1 complex, which is a structure-specific nuclease complex, and promotes the cleavage of its target DNA structures, including DNA replication and recombination intermediates [14]. These observations suggest that cellular proteins involved in replication stress or its repair contribute to Vpr-induced DDR induction.

The findings of this study revealed that Vpr-induced structural alteration of DNA lead to RPA70 loading and negative supercoil formations. We demonstrated lines of evidence supporting that such Vpr-induced structural alteration of DNA is an upstream event that leads to DDR induction as well as DSB formation. Similar activity was observed at the cellular level; when Vpr was artificially recruited onto a specific chromosomal locus, it induced RPA70 loading, accumulation of negative supercoils, formation of a Topo1-DNA covalent complex (Topo1-cc) and DSBs in the corresponding region. Notably, Vpr also induced the ubiquitination of histone H2B, thereby increasing histone mobility and promoting RPA70 loading onto the chromatin. Together with the data regarding the Vpr-induced promotion of viral DNA integration in resting macrophages in a Topo1-dependent manner, we proposed that the structural alteration of DNA by Vpr acts as a trigger event for DDR and DSBs induction, which ultimately contributes to HIV-1 infection.

## Results

### Vpr unwinds supercoiled DNA and alters its topological configuration

**Fig. 1:**
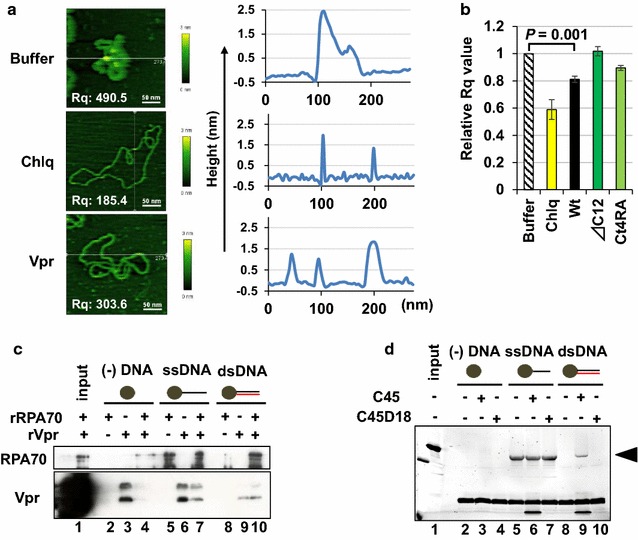
Vpr-induced structural alteration of DNA. **a** Representative AFM images of dsDNA and height profiles. Right panels depict height profiles detected in each cross section shown by white lines in the left panels. **b** rVpr decreased the Rq value. The experiments were repeated at least three times. Error bars indicate ± SEM. **c** RPA70 bound dsDNA after treatment of rVpr. RPA70 was pulled down using beads conjugated with DNA and detected by Western blotting (WB). **d** T4gp32 bound dsDNA after treatment with C45. T4gp32 was pulled down with DNA-bound beads in the presence of C45 or C45D18 peptide. Arrowhead, T4gp32

To elucidate the activity of Vpr on dsDNA, we first performed atomic force microscopy (AFM) to analyse the structural alteration of dsDNA. In these experiments, supercoiled dsDNA was incubated in a buffer solution with recombinant Vpr protein (rVpr), attached onto a freshly cleaved mica surface through a gentle Ni^2+^-mediated interaction, and then subjected to analysis [31]. AFM under liquid conditions can avoid potential artefacts resulting from the fixation or staining of DNA. Typical morphological changes and height profiles are shown in Fig. 1a. Markedly, treatment with chloroquine (Chlq), which is a DNA intercalator, resulted in the conversion of dsDNA into a highly expanded and de-condensed form (Fig. 1a, compare upper and middle panel and Additional file 1: Figure S1a). Interestingly, rVpr induced similar morphological changes in DNA (bottom panel). Each height profile was measured, and the root-mean-square (RMS) of the roughness (Rq) was calculated on the basis of the integrated data; Rq indicates the status and bulkiness of condensed dsDNA. As shown in Fig. 1b, the Rq value was significantly reduced by treatment with Chlq and rVpr (see also Additional file 1: Figure S1b). In contrast, *Δ*C12, which is a Vpr mutant that lacked the C-terminal 12 aa [16], did not affect the Rq value (Fig. 1b and Additional file 1: Figure S1b). Similarly, the Ct4RA mutant, in which all four arginines (R) of the RxRRxR motif of the C-terminal region were replaced with alanines (A), showed lower activity than Vpr-Wt (Fig. 1b and Additional file 1: Figure S1b, c). These data suggest that Vpr influences the structural alteration of dsDNA by interacting via positively charged residues in its C-terminal stretch. Notably, other Vpr mutants, including Q65R, R77Q and R80A, showed similar activity with Vpr-Wt (Additional file 1: Figure S1c).

Our initial experiment using AFM suggested that Vpr generates an ssDNA stretch in the dsDNA molecule. To confirm this, we tested whether RPA70 formed an association with dsDNA when treated with rVpr. As shown in Fig. 1c, Western blotting (WB) after a pull-down procedure detected RPA70 when dsDNA and RPA70 were incubated with rVpr (compare lanes 8–10). To further confirm the dependence of RPA70 loading on the C-terminal stretch of Vpr, we performed experiments using C45, which is a peptide made up of 45 aa of the C-terminal Vpr [32]. In this experiment, we used T4gp32 (T4 gene 32 protein), a well-established ssDNA-binding protein of the T4 phage [33, 34]. Consistent with the first experiment, we observed that T4gp32 bound dsDNA in the presence of C45 (Fig. 1d, lane 9). In contrast, no association was detected between dsDNA and T4gp32 when C45D18, which is a truncated form of C45 lacking the C-terminal 18 aa, was incubated (lane 10). Because Vpr did not interact with RPA70 or T4gp32 (Additional file 2: Figure S2a for RPA70 and Figure S2b for T4gp32), the data suggest that Vpr-induced unwinding of dsDNA occurred through its positively charged C-terminal region.

**Fig. 2:**
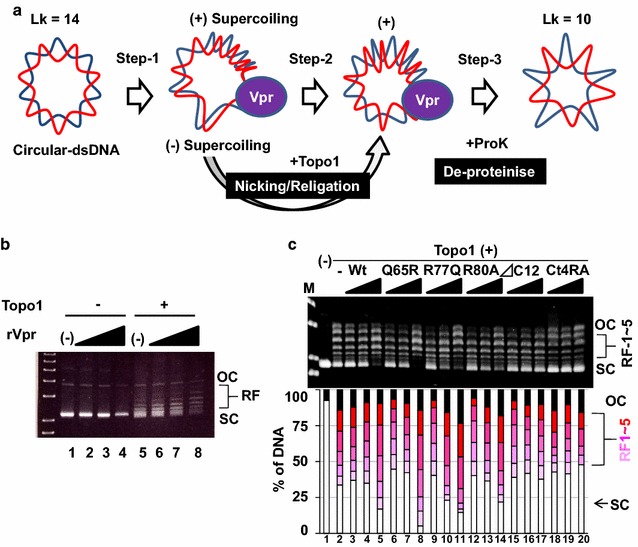
Vpr induced topological changes on DNA. **a** Schematic representation of experimental procedure for DNA supercoiling assay. Possible induction of partial unwinding of dsDNA by Vpr induces negative and positive supercoiling (Step-1). In the presence of *E. coli* Topo1, negative supercoiling (lower side) is relieved by nicking/relegation activity (Step-2). After the de-proteinisation, net amounts of linking number (Lk) are decreased (Step-3). **b** DNA supercoiling assay with rVpr-Wt. The effects of various amounts of rVpr were tested using *E. coli* Topo1. *OC* open circular, *RF* relaxed form, *SC* supercoiled. **c** DNA supercoiling assay with mutants of Vpr. Intensities of each topoisomer were quantified, and their relative amounts are shown in the bottom graph

When circular-dsDNA is relaxed in one part of a dsDNA, topological changes are aroused in the other (Fig. 2a, step-1). Such dsDNA with differential topologies can be detected using a DNA supercoiling assay [35], in which dsDNA is treated with *Escherichia coli* Topo1, followed by deproteinisation with proteinase K (ProK). On treatment with *E. coli* Topo1, negative supercoiling was removed through a nicking and re-ligation cycle (Fig. 2a, step-2), whereas treatment with ProK removed Vpr and Topo1, and generated relaxed forms of dsDNA (RFs) (Fig. 2a, step-3). Since agarose electrophoresis can separate each topoisomer, rVpr induced the formation of a slower-migrating species of DNA, representing RFs (Fig. 2b, lanes 6–8). Similar experiments were performed using various mutants of Vpr (Fig. 2c), indicating that Vpr-Wt increased the RFs of dsDNA (lane 5), whereas *Δ*C12 or Ct4RA did not (lanes 17 and 20). In contrast, Vpr mutants (Q65R, R77Q and R80A) showed activities comparable with that of Vpr-Wt (Fig. 2c, lanes 8, 11 and 14, respectively).

### Expression of Vpr induces the Topo1-cc formation

**Fig. 3:**
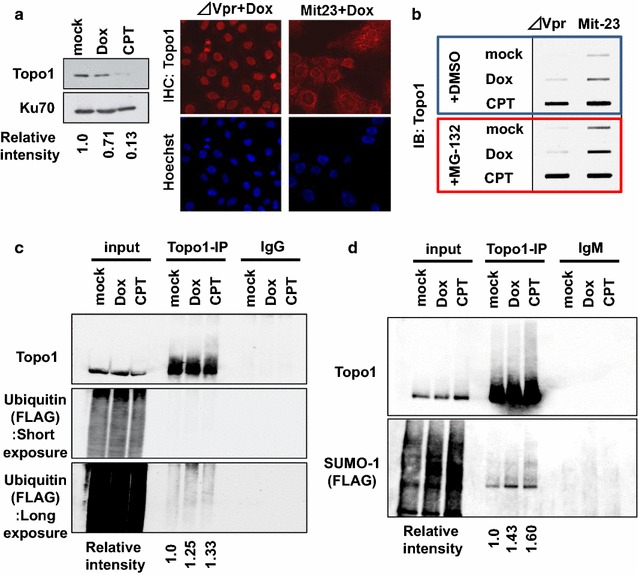
Vpr expression induces Topo1 stress. **a** Reduced expression of Topo1 under Vpr expression. Mit-23 cells were treated with Dox (3 μg/ml, 2 days) or CPT (20 μM, 1 h). By WB analysis, Topo1 and Ku70 as an internal control were detected. Relative intensities of Topo1 were calculated by normalizing the amounts of Topo1 by Ku70 (Left panel). Immunohistochemical analysis detected Topo1 and DNA (Hoechst-33258) (Right panel). *Δ*Vpr cells were used as a control cell line without exogenous Vpr. **b** Formation of Topo1-cc under Vpr expression. RADAR analysis was performed on Mit-23 and *Δ*Vpr cells, which were treated with Dox or CPT in the presence or absence of MG-132 (50 μM, 2 h). **c** Ubiquitination of Topo1 under Vpr expression. Mit-23 cells transfected with 3×FLAG/6×His-Ubiquitin were treated with Dox or CPT in the presence of MG-132, and immunoprecipitated with α-Topo1 antibody. Relative intensities of ubiquitinated Topo1 in the immunoprecipitates are shown. **d** Vpr induces SUMOylation of Topo1. Immunoprecipitation using α-Topo1 was performed in Mit-23 cells transfected with 3×FLAG/6×His-SUMO-1. CPT and Vpr expression induced modification of Topo1 with SUMO-1. Relative intensities of SUMOylated Topo1 in the immunoprecipitates are shown

Topo1-cc formation induces SSBs (DNA single-strand breaks) and DDR [27], while collision of a replication fork or transcription with the complex triggers DSB [27, 28]. To maintain genomic stability, Topo1-cc is removed by tyrosyl-DNA phosphodiesterase 1 (TDP1) and degraded by the SUMO/ubiquitin-mediated proteasomal degradation pathway [27, 36, 37]. For characterising the functional link between Vpr and Topo1 at the cellular level, we examined the Topo1 protein using Mit-23 cells, in which the tetracycline promoter tightly regulates Vpr expression [17]. When doxycycline (Dox) is added to these cells, Vpr is expressed at a level comparable with that in HIV-1 infected cells [38]. As observed in Fig. 3a, Vpr expression led to reduction in the level of Topo1 (relative level decreased to 0.71); an immunohistochemical analysis revealed that the nuclear signal of Topo1 was greatly reduced (Fig. 3a). Moreover, an increase in the level of Topo1-cc was detected by Rapid approach to DNA adducts recovery (RADAR) analysis, in which DNA–protein adducts were specifically recovered in the presence of chaotropic ion and detergents under denaturing conditions (Fig. 3b and Additional file 3: Figure S3a, b) [39]. Vpr-induced accumulation of Topo1-cc was enhanced by treatment with MG-132, which is a proteasomal inhibitor (Fig. 3b). Moreover, the Vpr expression increased the susceptibility of Topo1 to ubiquitination (Fig. 3c) and SUMOylation (Fig. 3d).

**Fig. 4:**
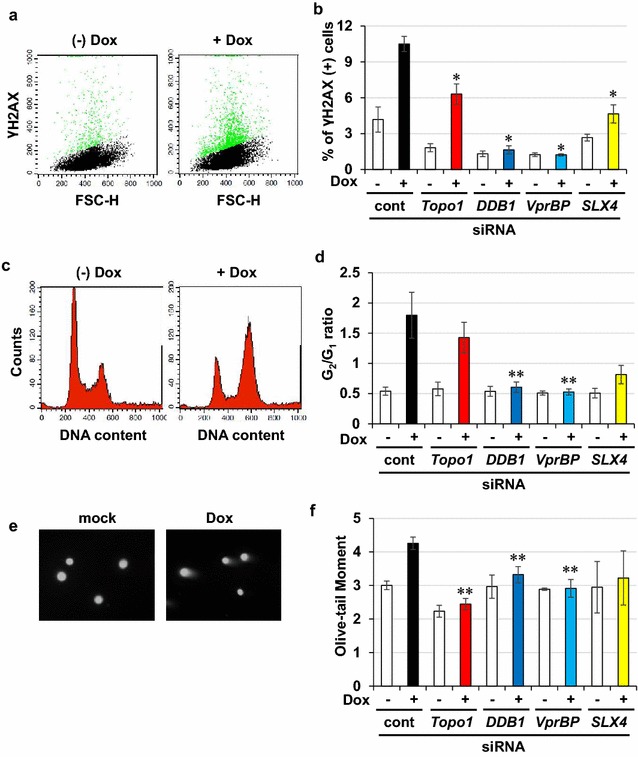
Vpr expression induces Topo1-mediated DDR and DSB.** a**,** b** Involvement of Topo1 in Vpr-induced DDR. Flow cytometry analysis was performed in Mit-23 cells transfected with indicated siRNAs to measure the Vpr-induced phosphorylation of H2AX. Representative scatter plots of γH2AX are shown in (**a**). Green colored plots were gated as γH2AX positive cells. Mean percentages of γH2AX positive cells are shown in (**b**). Error bars indicate ± SEM. Data obtained from more than three independent experiments. *P < 0.01. **c**, **d** Effects of *Topo1* siRNA on Vpr-induced cell-cycle abnormality. DNA contents were measured in Mit-23 cells transfected with indicated siRNAs by flow cytometry. Representative histograms of DNA contents (**c**) and average of ratios of G_2_ to G_1_ cells (**d**) are shown. **P < 0.05. **e**, **f** Involvement of Topo1 on Vpr-induced DSB. Neutral comet assay was performed in Mit-23 cells transfected with indicated siRNAs to quantitate the amounts of DSB. Representative images of each comet (**e**) and average of Olive-tail moment (**f**) are shown

To demonstrate the involvement of Topo1 in Vpr-induced DDR, we performed RNA interference (RNAi) experiment (knockdown efficiency is shown in Additional file 4: Table S1). Down-regulation of *Topo1* by siRNA reduced the number of γH2AX-positive cells (Figs. 4a, b, *P* = 0.007), whereas the over-expression of TDP1 decreased the number of Vpr-induced γH2AX-positive cells (Additional file 5: Figure S4a, b). Notably, down-regulation of *DDB1* and *VprBP* dramatically reduced cell-cycle arrest (Fig. 4c, d), whereas that of *Topo1* partially attenuated the Vpr-induced G_2_/M arrest. Because accumulation of Topo1-cc can lead to DSB formation [27, 28], we examined whether DSBs were induced in cells under Vpr expression. As shown in Fig. 4e, f, a neutral comet assay, which is a highly sensitive method to detect DSBs, revealed that Vpr induced DSB. In this case, the down-regulation of *Topo1* significantly attenuated Vpr-induced DSB (Fig. 4f).

### Forced accumulation of Vpr on a targeted DNA locus induces structural alteration of DNA and DDR

**Fig. 5:**
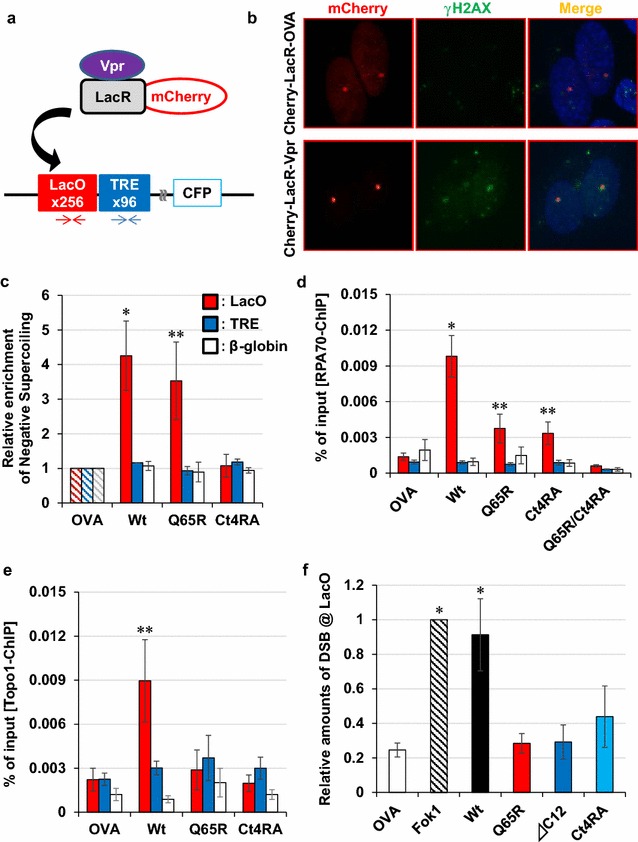
Forced accumulation of Vpr induces DDR in the targeted region. **a** Schematic of experiments using the LacO/LacR system. In U2OS/2-6-3 cells, 200 copies of p3216PECMS2β, which contains 256 copies of the LacO sequence and 96 copies of the tetracycline response element (TRE) upstream of the CFP coding region are integrated into a single site. In this cell lines, the LacR-Vpr fusion is forcibly recruited to the LacO repeat region (curved arrow). Arrows indicate PCR primers used for amplification of the LacO (red) and TRE (blue) regions (nucleotide sequence is shown in Additional file 20: Table S3). **b** Co-localised signals of Cherry-LacR-Vpr and DDR marker. Phosphorylated H2AX (γH2AX) was detected as a representative DDR marker after 2 days of transfection with indicated construct. **c** Formation of negatively supercoiled DNA in the Vpr-accumulated region. Two days after transfection, a psoralen-bound DNA was recovered and subjected to qPCR analysis with the primers shown in Fig. 5a. The relative copy numbers of LacO (red column), TRE (blue column), and β-globin (as a negative control; blank column) are shown. Error bars indicate ± SEM. *P < 0.01; **P < 0.05. **d** Enhanced loading of RPA70 by Vpr. Loading of RPA70 was quantitated by ChIP assay. **e** Formation of Topo1-cc by Vpr. ChIP assay was performed without cross-linking. MG-132 was treated for 2 h at 50 μM. **f** DSB induction by Vpr. LM-PCR was performed to measure the amounts of DSB-ends in the LacO repeat region. Each column indicates the amount of DSBs relative to LacR-fused Fok1 (positive control)

To investigate Vpr-induced structural alteration of DNA in the cell, we used U2OS/2-6-3, a human osteosarcoma cell line containing 200 copies of a construct composed of 256 *lac operator* (LacO) repeats at a single locus on 1p36 [40]. This cell line can be used to analyse the molecular events in a specific region of chromosomal DNA. When a fused protein of Vpr and Lac repressor (LacR) was expressed, Vpr was recruited to the LacO repeats (Fig. 5a) [40–42], which was confirmed by detecting mCherry-focus accumulated on a single distinct region in the nucleus (Fig. 5b, left panel). In these cells, mCherry-positive focus was precisely co-localised with all DDR-related molecules when a fused molecule of Cherry-LacR-Vpr was expressed: the following DDR-related molecules were examined: γH2AX (Fig. 5b, middle panels and Additional file 6: Figure S5a), phosphorylated ataxia telangiectasia mutated (ppATM-Ser1981), ppRPA32-Ser33, ppRAD17-Ser645 and FK2-stained mono- and poly-ubiquitin conjugates (Additional file 6: Figure S5b–e). On the other hand, Cherry-LacR fused Ovalbumin (OVA), which is a control molecule, generated no DDR signals overlapping with the mCherry-positive focus (Fig. 5b, upper panels and Additional file 6: Figure S5).

We next attempted to detect the structural alteration of DNA in the LacO repeats of U2OS/2-6-3 cells. For this purpose, we first used a psoralen, which is a DNA-intercalating cross-linker used for detecting regions containing negatively supercoiled DNA [43, 44]. Cells were incubated with a biotinylated psoralen and irradiated with UV to cross-link with DNA. Then, the psoralen-conjugated DNA fragments were recovered by a pull-down procedure with streptavidin beads. A qPCR analysis of the recovered DNA (Fig. 5a, primers shown as arrows below the boxes) revealed that the expression of LacR-fused Vpr-Wt increased the amount of psoralen-bound DNA corresponding with the LacO repeat region (Fig. 5c, *P* = 0.0058). Surprisingly, the Q65R mutant also increased psoralen binding to the LacO repeats (Fig. 5c, *P* = 0.034), whereas Ct4RA did not.

Further investigation of RPA70 loading on the LacO repeat region using a chromatin immunoprecipitation (ChIP) assay revealed that the expression of Cherry-LacR fused Vpr-Wt significantly increased RPA70 loading (Fig. 5d). In contrast, both Q65R and Ct4RA mutants increased RPA70 loading, but less potently than Vpr-Wt, which implies the dependence of RPA70 loading on the dual functional properties of Vpr; that is, DNA binding through the C-terminal region of Vpr and ubiquitination defective in the Q65R mutant. To test this notion, we monitored RPA70 loading with the Q65R/Ct4RA double mutant and observed that this mutant completely lost the ability to load RPA70 (Fig. 5d, *P* = 0.22). To evaluate the level of Topo1-cc, we recovered DNA without cross-linking under denaturing conditions, as in the RADAR analysis, and subjected the samples to ChIP assay done with the α-Topo1 antibody. Vpr-Wt significantly induced Topo1-cc accumulation on the LacO repeat region, whereas the Q65R and Ct4RA mutants did not (Fig. 5e, *P* = 0.049 for Vpr-Wt). As both Q65R and Ct4RA were severely defective in Topo1-cc accumulation, the coordinated functions of Vpr were required for provoking such downstream events.

We next quantified DSBs at the region where Vpr accumulated using a ligation-mediated (LM)-PCR (see Methods and Additional file 7: Figure S6) [41]. Surprisingly, Vpr-Wt induced DSBs at a level comparable with that by Fok1 endonuclease, which was used as a positive control (Fig. 5f). In contrast, the DSB level was not elevated in the Q65R and C-terminal mutants of Vpr. Requirement of Topo1 for Vpr-induced DSBs was confirmed also by a Cre/loxP-mediated Cherry-LacR-Vpr expression system, in which expression of Cherry-LacR-Vpr in U2OS/2-6-3 cells is induced by Cre expression (Additional file 8: Figure S7a, b). Obtained data again showed that down-regulation of *Topo1* suppressed Topo1-cc and DSB formation (Additional file 8: Figure S7c, d).

### Vpr-mediated chromatin remodelling is required for RPA70 loading

**Fig. 6:**
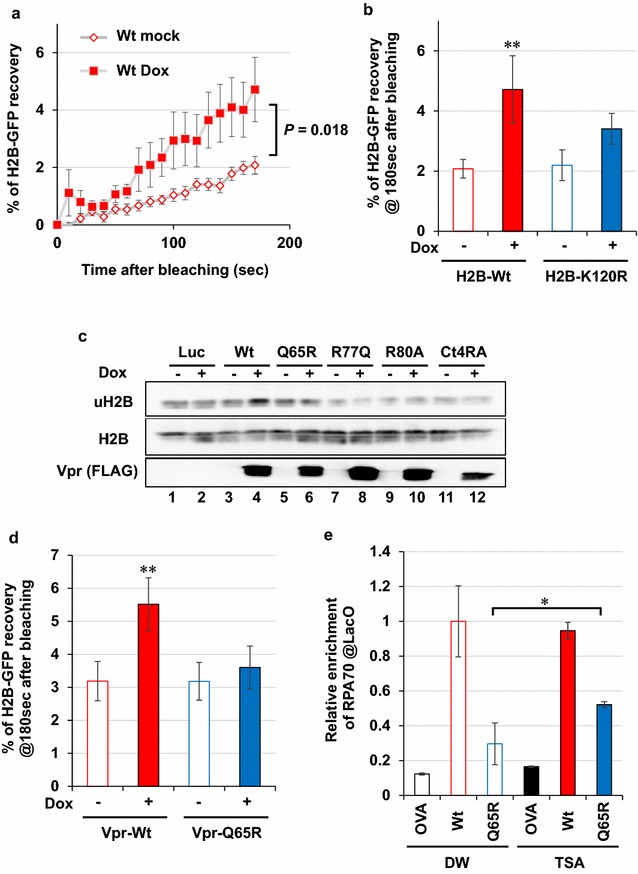
Vpr induces chromatin remodelling through histone H2B ubiquitination. **a** Increased mobility of H2B under Vpr expression. The FRAP assay was performed using Mit-23 cells with H2B-GFP. The collected values for the recovery rate of each GFP signal were subjected to statistical analysis. Error bars indicate ± SEM. **b** Vpr-induced mobilization of H2B depends on ubiquitination. Mean percentages of H2B-GFP recovery after 180 s of photo-bleaching were compared. **P < 0.05. **c** H2B was ubiquitinated by Vpr-Wt expression. For this experiment, we newly established cell lines (HT1080vRxt-Vpr), in which expression of Vpr-Wt (lanes 3 and 4), Vpr-Q65R (lanes 5 and 6), Vpr-R77Q (lanes 7 and 8), Vpr-R80A (lanes 9 and 10) and Vpr-Ct4RA (lanes 11 and 12) can be controlled by the tetracycline promoter, and ubiquitination of H2B on K120 was examined after Dox treatment (5 μg/ml, 2 days). As a negative control, a clone expressing Luciferase (Luc) was also included (lanes 1 and 2). Total H2B and Dox-induced expression of Vpr were detected. **d** Mobility of H2B was not increased by the Q65R mutant of Vpr. Graph shows mean percentages of H2B-GFP recovery after 180 s of photo-bleaching. **e** Defect of the Q65R mutant for RPA70 loading was rescued by TSA. After treatment with TSA (50 nM, 16 h), RPA70 loading onto the LacO repeats was quantitated by ChIP assay. *P < 0.01

For elucidating the functional link between Vpr-dependent ubiquitination and RPA70 loading, we measured the mobility changes of histone H2B using a fluorescence recovery after photo-bleaching (FRAP) assay [45]. Intriguingly, Vpr expression enhanced the recovery of H2B-GFP after photo-bleaching (Fig. 6a, *P* = 0.018). Moreover, this mobility change was reduced when H2B was mutated to a non-ubiquitinated form (K120R) (Fig. 6b, right column). Consistently, we observed higher levels of ubiquitination of H2B in cells expressing Vpr-Wt (Fig. 6c, lane 4), but not in cells expressing Q65R, R77Q, R80A and Ct4RA (Fig. 6c, lanes 6, 8, 10 and 12). Notably, the enhanced mobility of H2B-GFP was not detected in cells expressing the Q65R mutant (Fig. 6d, right column), implying that the reduction of RPA70 loading was caused by defective chromatin remodelling by Q65R mutant. Consistently, treatment with trichostatin A (TSA), a HDAC inhibitor that opens chromatin [46], successfully recovered RPA70 loading in the Q65R-expressing cells (Fig. 6e).

### Forced accumulation of Vpr induces proviral DNA integration at the targeted chromatin region

**Fig. 7:**
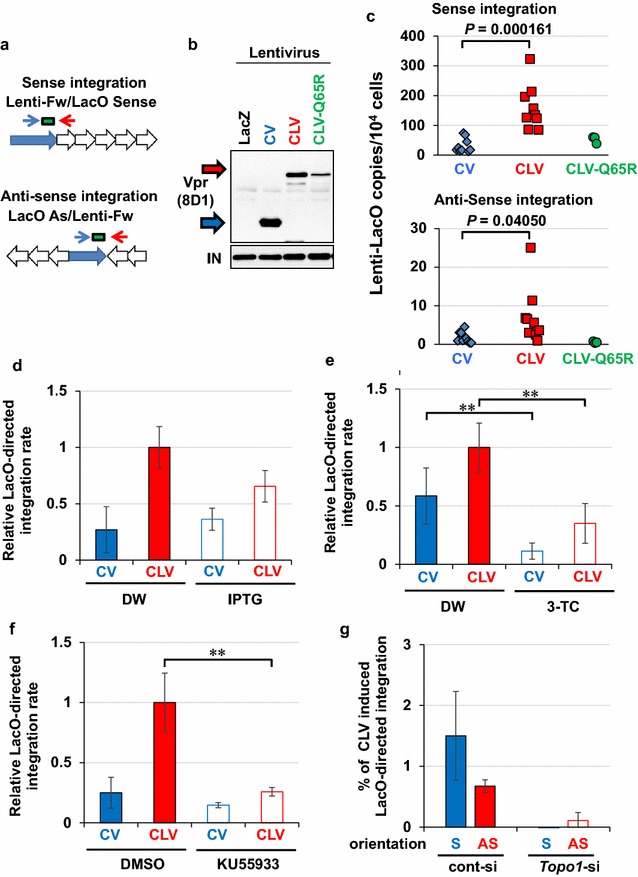
Proviral DNA integration in Vpr-accumulated sites. **a** Schematic of LacO-directed integration of proviral DNA. The qPCR analysis of the copy number of LacO-integrated proviral DNA was performed using PCR primers targeting the 3′-LTR (blue arrow) and LacO repeat (red arrow). The green box indicates the position of the TaqMan probe for the 3′-LTR (nucleotide sequence is shown in Additional file 20: Table S3). **b** Production of lentiviral particles with CV, CLV, and CLV-Q65R. Integrase (IN) was detected as an internal control. *LacZ*-coding lentivirus (LacZ) was included as a negative control. **c** Site-specific integration of proviral DNA. CV, CLV, and CLV-Q65R incorporated lentivirus was infected into U2OS/2-6-3 cells and subjected to qPCR analysis (Fig. 7a) at 2dpi (n = 9, 9, 3 for CV, CLV, and CLV-Q65R, respectively). Similar infectivity of each virus was confirmed by colony-formation assay (Additional file 12; Figure S11). **d** Effects of LacO/LacR inhibition on site-specific integration of proviral DNA. Cells were pretreated with IPTG (15 mM, 1 h) prior to the lentivirus infection. Error bars indicate ± SEM. Data were obtained from more than three independent experiments. **e** A reverse transcriptase inhibitor blocked the infection. Cells were pretreated with 3-TC (50 μM, 1 h) prior to lentivirus infection. **P < 0.05. **f** Site-specific integration of proviral DNA depended on ATM activity. ATM inhibitor, KU55933 (10 μM) was added to culture medium 1 h before infection. **g** Topo1 is important for site-specific integration of proviral DNA. A U2OS/2-6-3 subclone transduced with pCAL-loxP-CLV (263/loxP-CLV) was infected with Cre- or LacZ-expressing adenovirus for 2 days under down-regulation of *Topo1*. The cells were then infected with NL4-3/D64A/R− virus and subjected to qPCR analysis at 2dpi. The overall integration rate was quantitated by *Alu*-*gag* two-step nested qPCR (Additional file 11: Figure S10) to estimate the percentage of LacO-directed integration. *S* sense integration, *AS* anti-sense integration

To examine the association between Vpr-induced DSB and viral integration, we analysed HIV-1 integration in the Vpr-accumulated LacO region using qPCR (Fig. 7a and see “Methods” and Additional file 9: Figure S8). The frequency of proviral DNA integration in LacO repeats increased when LacR-fused Fok1 or Vpr-Wt was co-expressed at the time of HIV-1 infection (Additional file 10: Figure S9, Additional file 11: Figure S10). For demonstrating the direct effects of virion-associated Vpr, we prepared lentiviral particles composed of defective integrase (IN-D64A) and Cherry-LacR-fused Vpr (CLV) or Cherry-Vpr (CV) (Fig. 7b), infected them into U2OS/2-6-3 cells and performed qPCR. When CLV virus was infected, the frequency of proviral DNA integration into the LacO repeats significantly increased without marked effects on the overall viral infectivity (Fig. 7c and Additional file 12: Figure S11). In striking contrast, the infection of CLV-Q65R virus, a lentivirus with Cherry-LacR fused to Vpr mutant of Q65R, did not induce the LacO-directed integration, indicating that Vpr-induced ubiquitination and DSB is required for these integrations (Fig. 7b, c). The frequency of Vpr-induced LacO-directed integration was reduced by isopropyl β-d-1-thiogalactopyranoside (IPTG), which blocks LacO/LacR binding (Fig. 7d), as well as by the reverse-transcriptase inhibitor 2′,3′-dideoxy-3′-thiacytidine (3-TC) (Fig. 7e). In addition, these site-directed integrations were abrogated by the treatment with an ATM inhibitor (KU55933) (Fig. 7f), which is consistent with the results of a previous work showing that ATM activity is required for DSB-directed integration [8]. Moreover, RNAi experiment indicated that proviral DNA is integrated in the vicinity of Vpr-accumulated sites in a Topo1-dependent manner (Fig. 7g).

We also explored the lentiviral integration sites using linear amplification mediated (LAM)-PCR for estimating the frequency of selective integration into the LacO repeats (Additional file 13: Figure S12). Two out of 96 analysed samples (2%) contained LacO-dependent integrations in the genome.

### Topo1 requirement for Vpr-mediated enhancement of viral infection in non-dividing cells

**Fig. 8:**
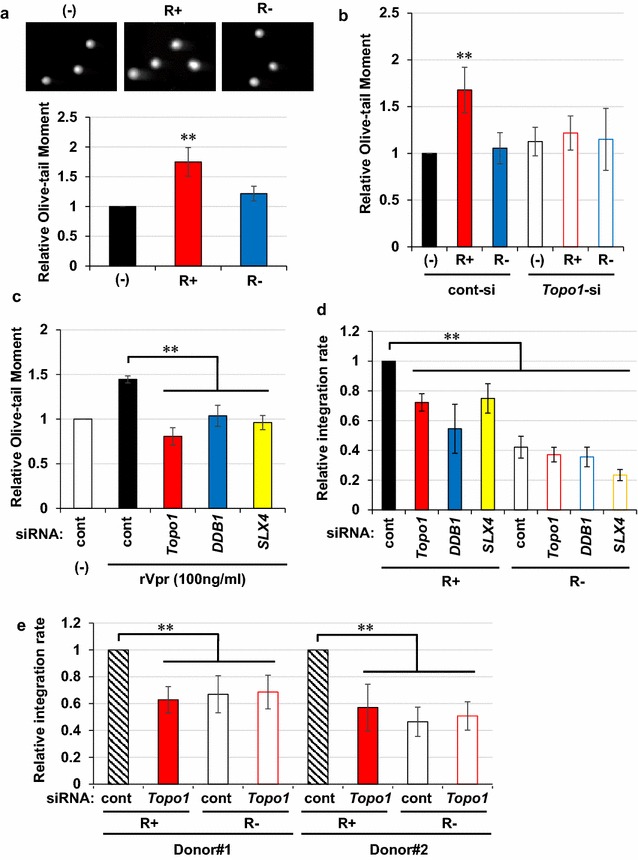
Vpr-induced DSB stimulates viral infection in resting macrophages. **a** Vpr induced DSBs in resting macrophages. Differentiated MM-6 cells were infected with NL4-3/D64A virus for 1 day, and then subjected to neutral comet assay. Representative images of each comet are shown (upper panel). The relative Olive-tail moment was evaluated (lower panel). Error bars indicate ± SEM calculated based on data obtained from more than three independent experiments. Data from non-infected cells (−), Vpr-proficient virus (R+), and Vpr-deficient virus (R−) are shown. **P < 0.05. **b** Involvement of Topo1 in Vpr-induced DSB. Neutral comet assay was performed as in **a** after 3 days of *Topo1* targeting siRNA transfection. **c** DSB induced by rVpr was blocked by down-regulation of *Topo1*. Three days after transfection of indicated siRNA, cells were treated with rVpr (100 ng/ml, 16 h). **d** Integration of proviral DNA was blocked by down-regulation of *Topo1*. MM-6 cells were infected with Vpr-proficient (R+) or deficient (R−) NL4-3 virus after transfection with siRNA, and the integration rate was quantitated by *Alu*-*gag* two-step nested qPCR at 2dpi; relative integration rates are shown. **e** Vpr-dependent increase of viral infectivity required Topo1 in MDMs. MDMs from two healthy donors were infected with Vpr-proficient (R+) or deficient (R−) NL4-3 virus 2 days after transfection with *Topo1* targeting siRNA, and the integration rates were quantitated as in **d** at 2dpi

Lastly, we examined the role of Vpr-induced DSB and Topo1 in the viral infection of resting macrophages. First, we prepared Vpr-proficient (R+) and deficient (R−) viruses, infected them into MonoMac-6 (MM-6) cells and applied the cells to neutral comet assay. In this experiment, we used IN-D64A mutant virus to exclude the effects of IN-dependent DSB [47, 48]. MM-6 is a monocyte leukaemia-derived macrophage-like cell line that can differentiate into resting macrophages following phorbol 12-myristate 13-acetate (PMA) treatment. A neutral comet assay revealed that infection of the R+ virus provoked DSBs in differentiated MM-6 cells (Fig. 8a). Furthermore, RNAi experiments revealed that DSB induction by the R+ virus was Topo1-dependent (Fig. 8b and Additional file 4: Table S1 for knockdown efficiency). Moreover, the addition of rVpr in the culture medium of MM-6 cells also induced DSBs that depended on *Topo1*, *DDB1* and *SLX4* (Fig. 8c). Notably, down-regulation of *Topo1* significantly suppressed the viral infectivity of the R+ virus, although the originally low infectivity of R− was unaffected (Fig. 8d, R+, *P* < 0.0001; R−, *P* = 0.78 and Additional file 14: Figure S13 for other sets of Topo1 siRNA). Vpr-dependent upregulation of viral infectivity was confirmed using luciferase assay (Additional file 15: Figure S14a) and EGFP expression (Additional file 15: Figure S14b). Similarly, down-regulation of *DDB1* and *SLX4* also led to decrease in the viral infectivity of the R+ virus. Finally, we performed similar experiments using primary monocyte-derived macrophages (MDMs) prepared from two healthy donors and confirmed that Vpr-dependent increase in viral infection depended on Topo1 (Fig. 8e and Additional file 4: Table S1 for knockdown efficiency).

## Discussion

Studies aimed at elucidating the mode of Vpr-induced DDR identified multiple Vpr-interacting cellular factors; however, the initial triggering event remains elusive. Here we presented evidence that structural alteration of DNA is the most upstream event of Vpr-induced DDR. Interestingly, our data suggest that structural alteration of DNA also induce DSB and contribute to HIV-1 infection in resting macrophages.

First, AFM observations revealed that supercoiled DNA shifted to a relaxed form when rVpr was added to DNA in an aqueous solution. This structural alteration was confirmed with the detection of negatively supercoiled DNA using the supercoiling assay with *E. coli* Topo1. Previous reports demonstrating that negative supercoiling of dsDNA occurs when RNA polymerase unwinds dsDNA or the nucleosome assembles on dsDNA [49, 50] together suggest that similar change in the topological configuration of dsDNA was induced by Vpr. This idea was also supported by Vpr-dependent loading of the ssDNA-binding proteins onto dsDNA.

Vpr-induced structural alteration of DNA was also observed in vivo in an experimental series using the LacO/LacR system in U2OS/2-6-3 cells. When a chimeric fusion protein of Vpr and LacR accumulated on the LacO region, we observed the formation of activated forms of multiple DDR-related molecules, chromatin loading of RPA70, accumulation of negative supercoiling, generation of Topo1-cc and DSBs in the same region foci (Fig. 5 and Additional file 6: Figure S5). RPA70 loading onto chromatin by Vpr-induced unwinding of dsDNA effectively explains the mechanism of Vpr-induced DDR.

**Fig. 9:**
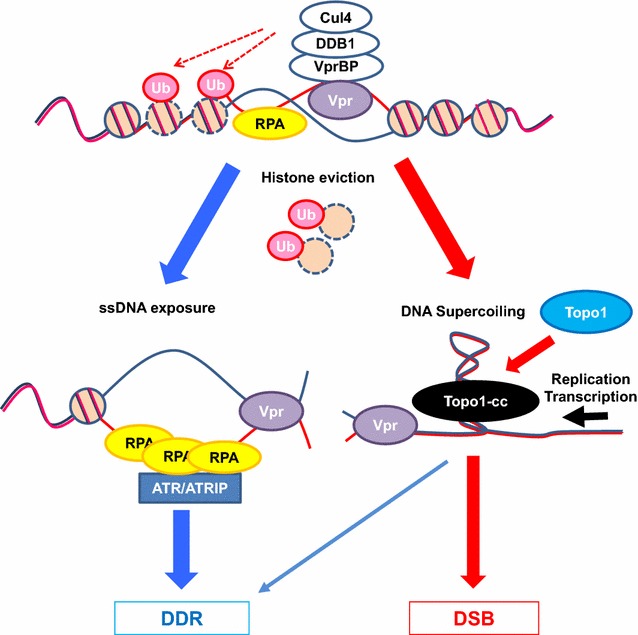
Hypothetical model of DDR and DSB induction by Vpr. Vpr unwinds dsDNA and allows limited loading of RPA70. Simultaneously, Vpr induces ubiquitination of histone H2B, and histone eviction occurs in the vicinity. Chromatin remodeling by histone eviction promotes efficient loading of RPA70, leading to G_2_/M checkpoint activation by ATR (left side). Vpr-induced unwinding of dsDNA in turn causes accumulation of supercoiling of DNA and formation of Topo1-cc (right side). In conjunction with DNA replication or transcription, Topo1-cc induces DSB formation, and proviral DNA is integrated at the DSB sites

Notably, our data suggested that Vpr-induced RPA70 loading was modulated at least two steps (Fig. 9). In the first step, the DNA-binding activity of Vpr changes the superhelicity of the DNA and partially unwinds dsDNA. This function was demonstrated using in vitro experiments (Fig. 1c, d), which also revealed the importance of the positively charged amino acids in the C-terminal stretch. In the second step, Vpr-mediated ubiquitination is required for chromatin remodelling, which enables the recruitment of cellular factors, including RPA70, to chromatin. This step was demonstrated via experiments using well-characterised Q65R mutant, which is defective in the ubiquitination process [21, 22]. Intriguingly, when the Q65R mutant was forced to accumulate on the LacO repeats, it could not stimulate RPA70 loading (Fig. 5d), although the RPA70 loading defect of the Q65R mutant was complemented by TSA (Figs. 5e, 6e). To elucidate this phenomenon, we investigated whether Vpr modulated chromatin remodelling and induced ubiquitination of histone H2B [51, 52]. Interestingly, the FRAP assay revealed that the recovery of H2B after photo-bleaching was more rapid in cells expressing Vpr-Wt than in those expressing the Q65R mutant. Similarly, a non-ubiquitinated H2B mutant exhibited reduced mobility in the Vpr-expressing cells. Moreover, the ubiquitination of H2B was promoted by Vpr-Wt, but not by the Q65R mutant. Considering that Cul4 regulates H2B ubiquitination for facilitating the DDR [53] and that the Q65R mutant is ubiquitination-defective due to its inability to bind DDB1/VprBP [21, 22], it is plausible that H2B is a target of Vpr-mediated ubiquitination, which is critical for Vpr-induced RPA70 loading. In addition to histone H2B, the association of Vpr with several chromatin modification factors, including p300, SNF2 h, NuRD and HDAC1 [38, 54–56], may also contribute to the efficient reorganization of chromatin. Our data suggested that the concerted actions of structural alteration of DNA and chromatin remodelling are required for efficient RPA70 loading by Vpr.

Notably, we found that Vpr-induced structural alteration of DNA could induce both DDR activation and DSB formation. In the latter process, Topo1 played a key role in creating DNA breaks (Figs. 4f, 8b, c; Additional file 8: Figure S7d), albeit its subtle contribution to Vpr-induced DDR activation (Fig. 4d). We also detected Vpr-induced DSB in resting macrophages in a Topo1-dependent manner (Fig. 8b, c), which suggested that Topo1-cc-mediated DSB could also arise in non-proliferating cells through interference with transcription [28]. Here, although we focussed on Topo1, we could not exclude the possibility of involvement of the other DNA structure modification factors such as Topo2, structure-specific nucleases including SLX4-Mus81/Eme1, and nucleotide excision repair (NER) factors in the processing of Vpr-induced aberrant DNA structures. Consistent with this possibility, we observed that the down-regulation of *SLX4* or *XPG* (a nuclease acting in NER) by siRNA suppressed Vpr-induced DSB (Fig. 4f and Additional file 16: Figure S15c, d). In addition, these factors also function in Topo1-mediated DSB induction. SLX4 causes DSBs through Topo1-bound DNA strand incision during the replication process [57, 58] and XPG induces DSBs through the processing of R-loops (DNA/RNA hybrids), which are formed when Topo1 inhibition blocks the transcription [59, 60]. In resting macrophages, it is plausible that R-loop formation is a major trigger event of Vpr-induced DSBs. Consistently, we observed an increase in R-loops at the Vpr-accumulated region (Additional file 17: Figure S16) and a decrease in Vpr-induced γH2AX-positive cells by the over-expression of RNaseH1, an RNase that degrades R-loops (Additional file 5: Figure S4).

We confirmed the requirement of SLX4 for Vpr-induced DDR, which was in concordance with the report of Laguette et al. [14], but in contrast with another study that reported SLX4 to be dispensable [61]. These discrepant observations are attributable to the different levels of residual activity of SLX4 under down-regulation by siRNA and gene disruption. In addition, differentially reorganised DNA repair pathway in SLX4 deficient cells leads to different cellular responses to Vpr.

Several lines of evidence indicate that DSBs increase the efficiency of HIV-1 infection, especially in resting cells [8, 18, 62, 63]. The finding that Vpr-induced DSBs are important for viral infection in resting macrophages effectively explains the previous observations that Vpr is required for the infection of resting cells [5–8]. Furthermore, the observation that DSB sites are directly targeted by proviral DNA integration supports the relevance of Vpr-induced DSB in viral infection [8].

The mechanism by which Vpr induces structural alteration of DNA remains unclear. In this study, we analysed DNA supercoiling using recombinant Vpr and Vpr-derived peptides, which suggested the necessity of C-terminal stretch of Vpr for DNA unwinding. Notably, we also detected that a higher concentration of Vpr-derived peptides (0.15–1.5 Vpr-peptides/bp) resulted in strongly underwound DNA (Additional file 18: Figure S17) in the DNA supercoiling assay. In a previous electron microscopic study, higher concentrations of Vpr (at least 4.5 Vpr-peptides/bp) induced the aggregation of DNA [26]. Because plectonemic or toroidal DNA structures are the compacted forms of negatively supercoiled DNA, it is possible that our current findings reflect the same phenomena as that reported in a previous study [26]. Siddiqui et al. [64] reported that Vpr-induced DDR required helical domain II (37–50 aa), but not the C-terminal region. These contradictory observations in the function of the C-terminal region of Vpr could be derived from the methods adopted for evaluating the Vpr-induced DNA damages. Siddiqui et al. [64] assessed the chromosomal abnormalities as Vpr-induced DNA damages, whereas we measured DSB itself by neutral comet assay and LM-PCR (Figs. 4f, 5f).

HIV treatment with cART can effectively suppress viral replication. However, complete eradication of HIV has not been successfully achieved, largely due to long-lived reservoir cells [1]. Recent observations indicate that viral sanctuary sites are distributed in various organs, including in the gut-associated lymphoid tissue and the central nervous system, in which persistent infections are observed [2, 3]. Vpr has reportedly been detected in the cerebrospinal fluid of HIV-positive patients [65, 66]. Notably, Topo1-mediated DNA damage is responsible for several neurodegenerative disorders [67], suggesting that Vpr may be involved in the development of HIV associated neurocognitive disorder [68] by exacerbating Topo1 insults. Our results provide a rationale for developing anti-Vpr compounds, which can significantly contribute to the improvement of the therapeutic regimen for HIV-positive patients under the current cART.

## Conclusions

We discovered that Vpr induces DNA structural alteration as an initial trigger of DDR and DSB. Notably, Vpr modulates chromatin remodelling by ubiquitinating histone H2B, and it facilitates RPA70 loading on unwound supercoiled DNA. We believe that our findings provide an answer to the long-standing question on how Vpr facilitates the infection of HIV-1 into macrophages.

## Methods

### Key resources (cell lines, chemicals, reagents, antibodies, recombinant DNA)

List of resources is included in Additional file 19: Table S2.

### Cell lines and cell culture

Cell lines used were MonoMac-6 (MM-6) (DSMZ), HEK293T (RIKEN Cell Bank), HT1080 (the Healthy Science Research Resources Bank), *Δ*Vpr, Mit-23 (derived from HT1080) [38], U2OS/2-6-3 (provided by Dr. David Spector, Cold Spring Harbor Laboratory, Cold Spring Harbor) [40], U2OS/2-6-3+pCAL-loxP-CLV (263/loxP-CLV), HT1080vRxt (retroviral RetroX-tet-ONE)-Luc or -Vpr, Mit-23+H2B-GFP and HT1080vRxt-Vpr+H2B-GFP. We confirmed no contamination of mycoplasma by periodically checking with Hoechst-33258 (Sigma-Aldrich) staining. MM-6 cells were cultured in RPMI 1640 with 10% fetal bovine serum (FBS) (Gibco) supplemented with 1% NEAA (Gibco) and 1% OPI media (Sigma). For differentiating into macrophage-like state, MM-6 cells were treated for 3 days with 50 nM of PMA. HEK293T and HT1080 cells were cultured in DMEM with 10% FBS, and *Δ*Vpr and Mit-23 cells were cultured in DMEM with 10% tetracycline free FBS (Hyclone) supplemented with HygromycinB (12.5 μg/ml) and G418 (100 μg/ml). U2OS/2-6-3 cells were cultured in DMEM with 10% FBS supplemented with HygromycinB (12.5 μg/ml). 263/loxP-CLV cells, U2OS/2-6-3 derived newly established cell line stably transduced with pCAL-loxP-CLV, were cultured in DMEM with 10% FBS supplemented with HygromycinB (12.5 μg/ml) and G418 (100 μg/ml). HT1080vRxt-Luc or -Vpr cells, HT1080 derived newly established cell lines stably transduced with retroviral RetroX-tet-ONE-Luc or -Vpr, were cultured in DMEM with 10% tetracycline free FBS supplemented with puromycin (1 μg/ml). All cell lines stably transduced with pEF-Bos-H2B-GFP (+H2B-GFP) were maintained in BlasticidineS (2 μg/ml) containing medium. All cells were grown at 37 °C in humidified atmosphere containing 5% CO_2_.

### Preparation of monocyte derived macrophages (MDMs)

Experimental procedures were approved by the internal review board. Peripheral blood mononuclear cells (PBMCs) of healthy volunteers who gave informed consent were isolated by Ficoll density gradient separation using Lymphoprep (Axis-Shield). Monocytes were isolated using monocyte isolation kit II (Miltenyi) by depletion of non-monocytes. For preparation of MDMs, isolated monocytes were cultured for 7 days in RPMI1640 supplemented with 10% FBS in the presence of 25–50 ng/ml of recombinant human macrophage-colony stimulating factor (rhM-CSF) (R&D).

### Bacterial strains

DH5α chemical competent *E. coli* (TOYOBO) was used for standard transformation. For the transformation of retrovirus, lentivirus, and HIV-1 vector DNA, Stbl3 chemically competent *E. coli* (Life Technologies) was used. In the case of protein expression, BL21DE3 (Stratagene) was used for transformation.

### rVpr purification from Wheat germ extract

By Wheat germ extract (WGE) cell free transcription/translation system (CellFree Sciences), FLAG-Strep-tag2 (F/S) fused to the N-terminus of Vpr (pNL4-3, GenBank: AF324493.2) was expressed by pEU-F/S-Vpr as template. After the protein synthesis, WGE was diluted in 3× volume of StA binding Buffer (50 mM Tris–Cl [pH 8.0], 500 mM NaCl, 1 mM EDTA, 1% NP40, 0.5% Brij-35) supplemented with RNaseA (100 μg/ml). After the incubation with Strep-Tactin agarose beads overnight at 4 °C on rotating platform, beads were washed with StA binding buffer, and eluted by 1×SA elution buffer (Calbiochem) supplemented with 1% NP40. The eluate was incubated with FLAG-M2 agarose beads overnight at 4 °C on rotating platform. The beads were extensively washed with phosphate buffer saline (PBS) (−), and eluted by 0.1 M HEPES [pH 2.5] and immediately neutralized by 1 M HEPES [pH 8.0], and adjusted to 50 mM of NaCl. The concentration of Vpr in the eluted fraction was determined by α-Vpr ELISA (MBL) [13] or Coomassie Brilliant Blue (CBB) gel staining compared to known amounts of Bovine serum albumin (BSA). The rVpr produced by WGE was used in experiments except Figs. 1c, 2c. Comparable activity of F/S-Vpr with rVpr from *E. coli* was confirmed by neutral comet assay.

### Purification of rVpr from *E. coli*

rVpr was purified as previously reported [13] with some modification. Briefly, BL21DE3 codon plus was transformed with pGEX-Vpr, in which Vpr was fused to C-terminus of GST followed by PreScission protease cleavage site, and cultured in 2×YTG supplemented with 100 µg/ml Ampicillin (2×YTG-Amp) over night at 30 °C. On the next day, the culture was inoculated to 1:50 ratio into fresh 2×YTG-Amp, and incubated at 37 °C until OD600 reached to 0.8. And then, 1 mM of IPTG was added to the bacterial culture medium, and further incubated for 3 h at 25 °C. Collected pellet from 500 ml culture was suspended in 20 ml of Binding buffer (20 mM sodium-phosphate buffer [pH 7.6], 150 mM NaCl, 0.5% TritonX-100, 10% glycerol) supplemented with 1 mM PMSF. After sonication and centrifugation, lysate was filtered through 5 μm filter and incubated with 500 μl of glutathione Sepharose (GSH) beads overnight at 4 °C on rotating platform. On the next day, beads were sequentially washed with Binding buffer supplemented with 500 mM NaCl or 1% TritonX-100, and Cleavage buffer (50 mM Tris–HCl [pH 7.0], 150 mM NaCl, 1 mM EDTA, 10% glycerol) twice, and then suspended in Cleavage buffer supplemented with 1 mM dithiothreitol (DTT) and 20 μl of PreScission protease. After overnight incubation on rotating platform at 4 °C, GSH beads were collected (Vpr remains bound to GSH beads), and washed with Binding buffer. Then, Vpr protein was eluted with Binding buffer supplemented with 0.1% TritonX-100. The eluate was incubated with new GSH beads to remove the un-cleaved GST-Vpr and PreScission protease for 1 h, and then supernatant was incubated with α-Vpr-antibody (8D1) conjugated CNBr-agarose beads overnight at 4 °C on rotating platform. The beads were extensively washed with PBS (−), and Vpr was eluted by 0.1 M HEPES [pH 2.5] and immediately neutralized by 1 M HEPES [pH 8.0]. The concentration of Vpr in eluted fraction was determined by α-Vpr ELISA [13]. The rVpr expressed in bacteria was used in experiments for Figs. 1c and 2c.

### rRPA70 purification

BL21DE3 was transformed with pET15-6×His-RPA70 (in which hRPA70 was tagged with hexa-histidine followed by PreScission protease cleavage site on N-terminus) and cultured in 2×YTG-Amp overnight at 30 °C. On the next day, the culture was inoculated to 1:50 ratio into fresh 2×YTG-Amp, and incubated at 37 °C until OD600 reached to 0.8. And then, 1 mM of IPTG was added to bacterial culture medium, further incubated overnight at 16 °C. Collected pellet from 500 ml culture was suspended in 20 ml of Buffer-A (50 mM Tris–HCl [pH 7.5], 500 mM NaCl, 0.5% TritonX-100, 10 mM mercaptoethanol, 10% glycerol) supplemented with 1 mM PMSF. After sonication and centrifugation, lysate was filtered through 5 μm filter and incubated with 10 ml of Affi-gel Blue resin (BioRad) overnight at 4 °C on rotating platform. Beads were washed with 20 ml of Buffer-A twice, and eluted with 10 ml of lysis buffer supplemented with 2.5 M NaCl. Eluate was diluted by 4-fold volume of Buffer-B (50 mM Tris–HCl [pH 8.0], 0.5% TritonX-100, 0.625% Empigen, 10% glycerol, 10 mM mercaptoethanol, 12.5 mM imidazole), followed by overnight incubation with Ni–NTA beads (Invitrogen). Beads were washed with Buffer-C (50 mM Tris–HCl [pH 8.0], 1 M NaCl, 0.5% Empigen, 0.5% TritonX-100, 10 mM mercaptoethanol, 10% glycerol, 10 mM imidazol) and Buffer-D (50 mM Tris–HCl [pH 8.0], 150 mM NaCl, 1% TritonX-100, 0.5% Empigen, 10% glycerol). Subsequently, beads were further washed with Buffer-E (50 mM Tris–HCl [pH 7.0], 150 mM NaCl, 0.1% Empigen, 10% glycerol) twice, and suspend in Buffer-E supplemented with 1 mM DTT, 1 mM EDTA, and 20 μl of PreScission protease. After overnight incubation on rotating platform at 4 °C, GSH beads were added to the eluate to remove the PreScission protease, and mixed for 1 h, and then supernatant was used as purified rRPA70 fraction. The concentration of RPA70 was estimated on CBB gel staining compared to known amounts of BSA.

### Sample preparation for AFM analysis

Fifty ng of pUC18 was incubated with 5 mM of Chlq or 0.5 μM of rVpr (1000-fold excess amount of Vpr to pUC18; 0.37 Vpr molecule/bp) in 50 μl of AFM reaction buffer (10 mM Tris–HCl [pH 8.0], 50 mM NaCl, 1 mM MgCl_2_) for 30 min at 37 °C. Prior to the binding of DNA, freshly cleaved mica were treated with AFM binding buffer (AFM reaction buffer supplemented with 5 mM NiCl_2_) for at least 10 min, and quickly blew out by N_2_ gas just before sample loading. And then, appropriate amounts of DNA sample (typically 10 μl) was dropped on the Ni^2+^-treated mica surface and placed for 10 min. Following quick washes of mica surface with AFM binding buffer twice, 100 μl of AFM binding buffer was loaded on mica for in liquid AFM observation.

### AFM measurement in aqueous solution

All measurements were carried out with a JPK NanoWizard ULTRA Speed AFM (JPK Instruments) on inverted microscope (IX71, Olympus) equipped with acoustic hood and active vibration isolation (Micro40, Accurion). Ultrashort cantilever (USC-F0.3-k0.3, NanoWorld) was used with 110–120 kHz drive frequency for high-speed, high-resolution imaging in liquid environment. The images were scanned in an intermittent contact mode (AC mode) for 2.0 μm × 2.0 μm area (512 × 512 pixels) at scan rate of 2.0 Hz, Z-range of 1.3 μm. Data processing was performed by JPK SPM Data Processing software (JPK Instruments). For processing images, the same parameters were used for all samples analysed in the same day. To obtain a root mean square of roughness (Rq) value, DNA molecule was manually surrounded by the minimal rectangle. For each treatment, at least 30 randomly selected DNA molecules were subjected to Rq value measurements, and independent experiments were performed more than three times.

### RPA70 Pull down assay by DNA-bound beads

At first, 10 pmol of biotinylated 80-mer ss/dsDNA was incubated with 10 μl of Dynabeads M280 Streptavidine (SA-beads) in SA binding buffer (10 mM Tris–HCl [pH 8.0], 1 M NaCl, 1 mM EDTA) at room temperature on rotating platform. After 15 min, beads were washed with SA binding buffer three times, and kept in IP buffer (50 mM Tris–HCl [pH 8.0], 150 mM NaCl, 1 mM EDTA, 1% NP40). Subsequently, 10 pmol of rRPA70 and rVpr was added to suspension of DNA-bound beads, and incubated for 1 h at 4 °C on the rotating platform. Beads were washed with IP buffer three times, and proteins recovered by pull-down procedures (pulled-down proteins) were analysed by WB.

### T4gp32 Pull down assay by DNA-bound beads

The beads bound with ss/dsDNA was prepared as above, and beads were suspended in 1×CutSmart buffer (NEB) supplemented with 1% NP40. Subsequently, 50 pmol of T4gp32 (NEB) and 100 pmol of C45 or C45D18 peptide was added to suspension of DNA-bound beads, and incubated for 1 h at room temperature on rotating platform. Beads were washed by 1×CutSmart buffer supplemented with 1% NP40 three times. Pulled-down proteins were separated on SDS-PAGE, followed by staining with Oriole fluorescent gel staining (BioRad), and visualized by LAS400 with UV transilluminator.

### Supercoiling assay

Supercoiled plasmid DNA, pBluescriptII (500 ng, Fig. 2b) or pUC18 (100 ng, Fig. 2c), was incubated in 20 μl of 1×CutSmart buffer with 0.025 U of *E. coli* Topoisomerase1 (NEB) and increasing amounts of rVpr (0.71, 2.36, 7.1 pmol and 0.63, 2, 6.32 pmol for Fig. 2b, c, respectively) for 30 min at 37 °C. After that, samples were heat-inactivated for 20 min at 80 °C, and incubated for 20 min at 55 °C with 0.5% SDS and Proteinase K (1 mg/ml) for de-proteinisation. Following phenol–chloroform extraction, purified DNA was separated on 0.8% agarose gel, and stained with ethidium bromide or 1×SyBr Gold (Life technologies) (Fig. 2b, c, respectively). Images were captured by GelDoc Ez (BioRad), and the intensity of each topoisomers was analysed by Image Lab software (BioRad).

### Immunohistochemistry (IHC)

Immunohistochemistry was performed as a standard protocol. Briefly, cells were fixed with 4% paraformaldehyde, and permeabilized with 0.5% TritonX-100. For the staining of Topo1, we performed pre-extraction with following buffer (20 mM HEPES [pH 7.5], 50 mM NaCl, 300 mM sucrose, 0.1% TritonX-100, 3 mM MgCl_2_) before fixing. After blocking with 5% skim milk/TBS with 0.1% Tween20, indicated antibodies were used. After that, optimal α-IgG antibody conjugated with Alexa fluorescent dye was used as a secondary antibody. Nuclei were stained by 1 μM of Hoechst-33258 and observed with fluorescent microscope (BX51, Olympus; BZ-X710, Keyence).

### DNA–protein covalent complex recovery (RADAR)

DNA–protein covalent complex was recovered according to a protocol previously reported [40]. Briefly, Mit-23 cells were treated with Dox (3 μg/ml) for 1 day or CPT (20 μM) for 1 h. In the case of MG-132 treatment, MG-132 (50 μM) was added to the culture medium 2 h prior to recovery. After treatment, cells were directly lysed in Buffer-M (6 M guanidine thiocyanate, 10 mM Tris–HCl [pH 6.5], 20 mM EDTA, 4% TritonX-100, 1% *N*-lauroylsarcosine, 1% DTT), and ethanol precipitation was performed by addition of half volume of 99% ethanol and centrifugation. Following washing with 70% ethanol twice, pellets were dissolved and sonicated in 8 mM NaOH solution. Amounts of DNA were quantitated by Picogreen (Invitrogen) with InfiniteM1000 PRO plate reader. For slot-blotting, equal amounts of DNA were absorbed to nitrocellurose membrane (BioRad) by BioDot-SF microfiltration apparatus (BioRad) with TBS buffer (10 mM Tris [pH 7.5], 150 mM NaCl). Topo1-cc or dsDNA were detected by respective antibodies.

### Immunoprecipitation (IP)

Immunoprecipitation for detecting post-translational modifications of Topo1, Mit-23 cells were transfected with 3×FLAG/6×His-tagged ubiquitin or SUMO-1 expression vector. On the next day, cells were suspended in medium with or without of Dox (3 μg/ml) and incubated for 1 day. Then, MG-132 (50 μM) was added to all samples for 2 h. For CPT-treatment, cells were treated for 1 h before recovery. For detecting the ubiquitination, cells were lysed in 0.5×RIPA buffer (50 mM Tris–HCl [pH 8.0], 150 mM NaCl, 1 mM EDTA, 1% NP40, 0.25% sodium deoxychorate (DOC), 0.05% SDS) supplemented with 1× protease inhibitor cocktail (Roche) and 10 mM *N*-ethylmaleimide (NEM), and subjected to sonication. After that, samples were incubated with 50 U of Benzonase and 2.5 mM MgCl_2_ for 1 h at 16 °C. Following centrifugation, equal amounts of supernatant were incubated for 16 h with α-Topo1 polyclonal antibody or Rabbit-IgG at 4 °C. Immunocomplex was recovered by DynaBeads ProteinA and washed with 0.5×RIPA buffer, and subjected to WB analysis. For detecting SUMO-1 modification, cells were boiled in Hot-Lysis buffer (10 mM Tris–HCl [pH 8.0], 150 mM NaCl, 2% SDS) supplemented with 1× protease inhibitor cocktail and 10 mM NEM for 10 min, and diluted in 2-fold volume of Hot-Lysis Dilution buffer (10 mM Tris–HCl [pH 8.0], 150 mM NaCl, 1 mM EDTA, 1% TrironX-100) supplemented with 10 mM NEM. After the sonication, samples were incubated for 1 h with 50 U of Benzonase and 2.5 mM MgCl_2_ at 16 °C. Following centrifugation, samples were further diluted in 19-fold volume of Hot-Lysis Dilution buffer (at this time concentration of SDS was 0.05%), and equal amounts of supernatant were incubated for 16 h with α-Topo1 monoclonal antibody or Mouse-IgM at 4 °C. Immunocomplex was recovered by ProteinL magnetic beads (Thermo Scientific) and washed with Hot Lysis Wash buffer (10 mM Tris–HCl [pH 8.0], 250 mM NaCl, 1 mM EDTA, 1% NP40), and subjected to WB analysis.

### Flow cytometry (FCM) analysis

Two days before Dox treatment, Mit-23 cells were transfected with indicated siRNA (50 nM) or plasmid DNA by Lipofectamine RNAiMax or Lipofectamine 2000 transfection reagent, respectively. After culturing cells in the presence of Dox (3 μg/ml) for 1 day, we harvested cells and suspended in freshly prepared 70% ethanol. Recovered cells were kept for 2 h at −20 °C. After that, cells were suspended in FCM buffer [PBS (+) supplemented with 4% FBS and 0.1% TritonX-100] and left at 4 °C for 30 min. For γH2AX-staining, cells were incubated with 100 μl of FCM buffer supplemented with 0.5 μg of α-γH2AX-antibody FITC conjugated (Millipore). After incubation for 2 h at 4 °C, samples were washed three time with FCM buffer, and then nuclear staining was achieved by propidium iodide (1 μg/ml)/PBS (−) supplemented with RNaseA (200 μg/ml). After treatment for 1 h at room temperature in the dark place, FCM analysis was performed by FACSCalibur on at least 10,000 cells.

### Neutral comet assay

Neutral comet assay was performed following the manufacturer’s instruction (Trevigen) with some modification. Briefly, Mit-23 cells were harvested by 2 mM EDTA/PBS (−). Differentiated MM-6 cells were detached by Accutase (Innovative Cell Technologies) and collected. After resuspension in PBS (−), cells were embedded in Low-Melting Agarose (Trevigen), spread and solidified over the Comet Slides (Trevigen) on ice. The slides were immersed in Lysis Buffer (Trevigen) for at least 1 h, and then incubated in neutral electrophoresis buffer (0.1 M Tris-Ac [pH 9.0], 0.3 M NaOAc·3H_2_O) for 30 min at 4 °C. After electrophoresis for 1 h at 0.75 V/cm at 4 °C, samples were fixed in precipitation buffer (1 M NH_4_Ac, 85% ethanol) and 70% ethanol. After staining nuclei by SyBr gold, images were captured by (BZ-X710, Keyence) with 20× objectives utilizing function of Z-stacks and image stitching for obtaining with high resolution image of large field. Integrated images were analysed by Comet Assay IV software (Perceptive Instruments) using “Olive-tail moment” as a parameter of extent of DSB. Olive-tail moment generally indicate the extent of DNA damages, which calculated by product of the tail length and the fraction of total DNA in the tail. In Comet Assay IV software (Perceptive Instruments), “Olive-tail moment” is defined as: the product of the proportion of tail intensity and the displacement of tail center of mass relative to the center of the head. In all experiments, at least 80 nuclei were subjected to analysis.

### Pull-down assay of negative supercoiled DNA

U2OS/2-6-3 cells were transfected with indicated plasmid DNA by Viafect transfection reagent (Promega). After 2 days of transfection, 5 × 10^6^ cells were suspended in 250 μl of RSB (10 mM Tris [pH 7.5], 10 mM NaCl, 3 mM MgCl_2_) and combined with 3.5 ml of RSB with 0.1% NP40. After gentle mixing, cells were centrifuged at 500×*g* for 10 min at 4 °C. Nuclear pellet was suspended in PBS (−) supplemented with 5 μM of Ez-Link Psoralen-PEG_3_-Biotin (bPso) (Thermo scientific) for 30 min at 4 °C. After centrifugation at 500×*g* at 4 °C for 10 min, pellet was suspended in 100 μl of PBS (−) and removed to 96-well plate. For cross-linking the bPso to DNA, 365 nm wavelength of UV (UVL-21, UVP) was irradiated for 30 min on ice, and then collected nuclei were lysed in sonication buffer (50 mM Tris [pH 7.5], 140 mM NaCl, 1 mM EDTA, 1 mM EGTA, 1% TritonX-100, 0.1% DOC, 0.1% SDS) supplemented with 1× protease inhibitor cocktail. To obtain the DNA fragment with average size of 250 bp, sonication was carried out following conditions: 20 cycles of 30 s-ON/30 s-OFF, using Bioruptor UCD-250 (Cosmo Bio). After that, samples were centrifuged at 16,000×*g* for 30 min at 4 °C, and obtained supernatants was gently mixed with SA-beads blocked with 0.5% BSA and 100 μg/ml salmon sperm DNA at least 12 h. SA-beads were sequentially washed with RIPA buffer (50 mM Tris–HCl [pH 8.0], 150 mM NaCl, 1 mM EDTA, 1% NP40, 0.5% DOC, 0.1% SDS) for 10 min, High salt wash buffer (50 mM Tris–HCl [pH 8.0], 500 mM NaCl, 1 mM EDTA, 1% NP40, 0.5% DOC, 0.1% SDS) for 15 min, LiCl wash buffer (50 mM Tris–HCl [pH 8.0], 250 mM LiCl, 1 mM EDTA, 1% NP40, 0.5% DOC) for 15 min, and 1×TE buffer (10 mM Tris–HCl [pH 8.0], 1 mM EDTA) for 10 min twice. All processes were performed at 4 °C on rotating platform. After washing, SA-beads was treated with RNaseA (1 mg/ml) in 1×TE buffer for 30 min at 37 °C, and ProteinaseK (100 μg/ml) with 0.5% SDS for 1 h at 55 °C. And then supernatant was purified by phenol–chloroform extraction (fraction-1). Furthermore, residual DNA on SA-beads was further extracted by 95% formamide with 10 mM EDTA for 10 min at 90 °C (fraction-2). Combined fractions were purified by ethanol precipitation, and purified DNA was dissolved in 1×TE buffer and subjected to qPCR analysis with SyBr Premix ExTaq Tli RNaseH plus (TaKaRa) by StepOne Real-time PCR system. Oligonucleotides used in this procedure are listed in (Additional file 20: Table S3).

### Chromatin immunoprecipitation (ChIP)

U2OS/2-6-3 cells were transfected with indicated plasmid DNA by Viafect transfection reagent. After the 2 days of transfection, cells were cross-linked with 1% paraformaldehyde for 10 min, followed by quenching with 0.125 M glycine for 5 min at room temperature. After washing with PBS (−) twice, cells were suspended in Buffer-1 (10 mM HEPES [pH 7.5], 10 mM EDTA, 0.5 mM EGTA, 0.75% TritonX-100) supplemented with 1×protease inhibitor cocktail and incubated for 10 min on ice. After the centrifugation at 1700×*g* for 10 min at 4 °C, collected cells were resuspended in Buffer-2 (10 mM HEPES [pH 7.5], 200 mM NaCl, 1 mM EDTA, 0.5 mM EGTA) supplemented with 1× protease inhibitor cocktail and incubated for 5 min on ice. Subsequently, cells were recovered by centrifugation (1700×*g* for 10 min at 4 °C), and lysed in SDS lysis buffer (50 mM Tris–HCl [pH 8.0], 10 mM EDTA, 1% SDS) supplemented with 1× protease inhibitor cocktail. After fragmentation to average length of 250 bp, samples were centrifuged at 16,000×*g* for 30 min at 4 °C, and obtained supernatants were diluted in 9-fold volume of ChIP Dilution buffer (50 mM Tris–HCl [pH 8.0], 167 mM NaCl, 1.1% TritonX-100, 0.11% DOC). After pre-clearing with ProteinG Sepharose beads and IgG Sepharose beads, 2 μg of α-RPA70 monoclonal-antibody or mouse-IgG were added to each aliquot of chromatin lysate, and incubated on rotating platform for over 12 h at 4 °C. Immunocomplex was recovered by Dynabeads ProteinG blocked with 0.5% BSA and 100 μg/ml salmon sperm DNA. After 1 h incubation, beads were extensively washed as described in “Pull-down assay of negative supercoiled DNA”, and incubated for 6 h at 65 °C in 1×TE buffer supplemented with 250 mM NaCl and 0.5% SDS, and treated with RNaseA (1 mg/ml) for 30 min at 37 °C, and ProteinaseK (100 μg/ml) for 1 h at 55 °C. At this time, input DNA was treated as same way. For eluting DNA, beads were incubated with 0.1 M NaHCO_3_ and 1% SDS for 30 min at 65 °C, and recovered DNA was purified by phenol–chloroform extraction and ethanol precipitation. Purified DNA was dissolved in 1×TE buffer and subjected to qPCR analysis. Oligonucleotides used in this procedure are listed in (Additional file 20: Table S3).

### Native-ChIP

For detecting Topo1-cc (covalent complex of Topo1 and DNA), ChIP assay without cross-linking was performed. U2OS/2-6-3 cells were transfected with indicated plasmid DNA by Viafect transfection reagent. After 2 days of transfection, cells were treated with MG-132 (50 μM) for 2 h, and directly lysed in Buffer-M. DNA–protein covalent complex was recovered by ethanol precipitation as described. Pellets were dissolved in SDS lysis buffer supplemented with 1× protease inhibitor cocktail and sonicated. Following dilution, immunoprecipitation was performed with α-Topo1 polyclonal-antibody or Rabbit IgG. Immunocomplex was recovered by DynaBeads ProteinA blocked with 0.5% BSA and 100 μg/ml salmon sperm DNA, and washed as with the ChIP protocol. Collected beads were incubated in 1×TE with RNaseA (1 mg/ml) for 30 min at 37 °C, ProteinaseK (100 μg/ml) with 0.5% SDS for 1 h at 55 °C, and 0.1 M NaHCO_3_ with 1% SDS for 30 min at 65 °C. Eluted DNA was purified, and analysed by qPCR as in standard ChIP assay. Oligonucleotides used in this procedure are listed in (Additional file 20: Table S3).

### DNA/RNA hybrids immunoprecipitation (DRIP)

Genomic DNA was purified by DNeasy Blood & Tissue Kit (Qiagen) or Buffer-M according to RADAR protocol [39]. Following sonication, samples were treated with or without of RNaseH (20 U, NEB) in 1×RNaseH Reaction buffer overnight at 37 °C. After purification, DNA pellets were dissolved in DRIP buffer (50 mM Tris–HCl [pH 8.0], 150 mM NaCl, 1 mM EDTA, 0.05% TritonX-100), and incubated with S9.6 antibody (KeraFast) or mouse IgG for 12 h at 4 °C on rotating platform. Following processes were performed as with Native-ChIP, and analysed by qPCR as in standard ChIP assay. The promotor and terminator regions of *Actin* gene were analysed by the qPCR analysis, as negative and positive control of DRIP assay, respectively [69]. Oligonucleotides used in this procedure are listed in (Additional file 20: Table S3).

### Ligation-mediated (LM)-PCR

LM-PCR experiments were performed according to a protocol previously reported [41] with some modifications. In brief, after 2 days of transfection, cells were directly lysed in HMW buffer (10 mM Tris–HCl [pH 7.5], 100 mM NaCl, 1 mM EDTA, 0.5% SDS) supplemented with ProteinaseK (200 μg/ml), and incubated overnight at 55 °C without agitation. After phenol–chloroform extraction, same volume of isopropanol was added to sample, and DNA was gently picked up and rinsed with 70% ethanol. Recovered DNA was suspended in 1×TE supplemented with RNaseA (100 μg/ml). Equal amounts of DNA was treated with Quick-Blunting Kit (NEB) and ligated with blunt-end linkers (JW-Linker: annealed JW102 with JW103). Following purification, equal amounts of DNA were subjected to qPCR analysis with LacO-Rev and JW102 primers using SyBr Premix ExTaq Tli RNaseH plus. For estimating the amounts of LacO/JW-linker junction, corresponding DNA fragment was cloned into pMD20, and used as standard sample. The qPCR against to *β*-*globin* was carried out in parallel to normalize the amounts of input DNA. Oligonucleotides used in this procedure are listed in (Additional file 20: Table S3).

### Fluorescence recovery after photobleaching (FRAP) assay

For FRAP assay, newly established cell lines: Mit-23 with H2B(Wt)-GFP or H2B(K120R)-GFP, HT1080vRxt-Vpr-Wt or -Q65R with H2B(Wt)-GFP, in which pEF-Bos-H2B-GFP was stably transduced to Mit-23 or HT1080vRxt-Vpr, were treated with Dox (3 μg/ml) for 1.5 days. Images were obtained by a Leica TCS SP5 confocal microscope with a Leica HCX APO 100×/1.40–0.60 oil immersion lens, and obtained data were analysed by Leica LAS AF Lite software (Leica). For GFP excitation, we used the 488 nm line of an Argon laser and fluorescence emission was collected between 500 and 530 nm. All experiments were done on warmed stage at 37 °C. In experiments, pre-bleach image was acquired by three consecutive images. Then a single square on the nucleus was bleached with five times of laser pulses of 1.318 s at 100% power. Images were then collected at 10 s intervals for 180 s. For calculating the rate of FRAP, the background signals (ROI^B^) were subtracted from the region of interest for bleached area (ROI^+^) and un-bleached area (ROI^−^). The corrected signal of ROI^+^ was normalized by ROI^−^ at each time point, and relative signal intensity compared to pre-bleaching (ROI^0^) was used to evaluate the change of GFP intensity, according to the equation;

$${\text{Relative}}\;{\text{signal}}\;{\text{intensity}} = \{ ({\text{ROI}}^{ + } - {\text{ROI}}^{\text{B}} ) \div ({\text{ROI}}^{ - } - {\text{ROI}}^{\text{B}} )\} \div ({\text{ROI}}^{0} - {\text{ROI}}^{\text{B}} )$$

The recovery of GFP signal intensity was expressed by subtracting the relative signal intensity of immediately after bleaching from that of each time point. In all experiments, at least 10 cells were subjected to analysis.

### Production of pseudotyped HIV-1

For production of Vesicular Stomatitis Virus Glycoprotein (VSV-G) pseudotyped HIV-1, HEK293T cells were co-transfected with pNL4-3/E- and pHIT-VSV-G using FuGENE6 transfection reagent (Promega). On the next day, culture medium was changed to DMEM supplemented with 0.1% FBS. On the day 2 after transfection, culture supernatant containing the virus were recovered and filtered through a 0.45 μm-filter, and viral titer was determined by p24 ELISA kit (Zeptometrics). NL4-3-Luc/E- and NL4-3-EGFP/E-, which contains *Luciferase* and *EGFP* in-frame of *Nef*, were prepared as same manner. Viral samples were treated with DNase I (Takara) before infection.

### Lentivirus production

Lentivirus was produced using Virapower Lentiviral packaging mix (Invitrogen) according to a manufacturer’s instruction with some modification. Briefly, HEK293T cells were co-transfected with pLenti6, pLP1-D64A, pLP2, and pLP-VSV-G using Lipofectamine 2000 transfection reagent. Culture medium was replaced to fresh medium at 16 h after transfection. On the day 2 after transfection, culture supernatant containing the virus was filtered through a 0.45 μm-filter and ultra-centrifuged at 40,000 rpm for 1 h at 4 °C (Optima TLX ultracentrifuge, Beckman Coulter). After removing supernatant, viral pellet was dissolved in Opti-MEM (Life technologies), and viral titer was determined by p24 ELISA kit. Viral samples were treated with DNase I before infection.

### HIV-1 infection to MM-6 cells

For pseudotyped HIV-1 infection, 2.5 × 10^5^ MM-6 cells were differentiated by 50 nM of PMA for 3 days in 12-well plate. In RNAi experiments, cells were transfected with 300 nM of indicated siRNA by Nucleofector Kit-V and suspended in PMA containing medium. After 3 days, NL4-3 viral solution (p24, 50 ng) was added, and incubated for 2 h. Following washing twice with pre-warmed medium, cells were cultured in PMA containing medium for additional 1 or 2 days, for neutral comet assay or *Alu*-*gag* two-step nested qPCR analysis, respectively. For luciferase assay, cell cultures (50,000 cells/well, in 96well plate) at 3 days post-infection (dpi) were directly lysed in equal volume of One-Glo Luciferase assay system (Promega) solution, and relative light units (RLU) were measured by microplate luminometer (Veritas). For FCM analysis of EGFP, cells were fixed with 1% paraformaldehyde at least 30 min at room temperature at 3dpi, and analysed by FACSCalibur on at least 10,000 cells.

### HIV-1 infection to MDMs

For pseudotyped HIV-1 infection, 2.5 × 10^5^ MDMs were differentiated by 25–50 ng/ml of rhM-CSF for 5 days in 12-well plate. In RNAi experiments, cells were transfected with 50 nM of indicated siRNA by Lipofectamine RNAiMax transfection reagent for 4 h and replaced to rhM-CSF containing medium. After 2 days of transfection, NL4-3 viral solution (p24, 12.5 ng) was added, and incubated for 2 h. Following washing twice with pre-warmed medium, cells were cultured in rhM-CSF containing medium. *Alu*-*gag* two-step nested qPCR analysis was performed at 2dpi.

### HIV-1 infection to U2OS/2-6-3 cells and analysis of LacO-directed integration

U2OS/2-6-3 cells of 5 × 10^5^ were transfected with indicated plasmid DNA by Viafect transfection reagent. After 2 days of transfection, cells were incubated with NL4-3 viral solution (p24, 100 ng) for 2 days, and subjected to qPCR analysis. Genomic DNA was extracted using Quickgene Nucleic acid isolation system (KURABO). The qPCR was done with LacO-(sense or antisense), Lenti-5237F, and TaqMan probe (pLenti6-LTR probe) using SsoAdvance universal probe Supermix by StepOne Real-time system. To measure copy numbers of integrated viral DNA that possessed the LacO/proviral DNA junction, corresponding DNA fragment was cloned into pMD20 and used as standard samples. In parallel, qPCR for *β*-*globin* was performed for normalization of input DNA. At this time, *Alu*-*gag* two-step nested qPCR was performed and measuring the overall infectivity. Oligonucleotides used in this procedure are listed in (Additional file 20: Table S3).

### *Alu*-*gag* two-step nested qPCR

Genomic DNA was extracted using Quickgene Nucleic acid isolation system, and 100 ng of DNA was subjected to 12 cycles of *Alu*-*gag* 1st PCR using AmpliTaq gold 360 with Alu-F/R and 1st-gag-R. And then, qPCR was performed with 2-LTR-S and 2nd tag-R, and TaqMan probe (Probe-2). For determining the frequency of the integration, gDNA containing 0.485 copies of HIV integration/cell [8] was subjected to amplification at the same time. The qPCR against to *β*-*globin* was carried out in parallel to normalizing the amounts of input DNA. Oligonucleotides used in this procedure are listed in (Additional file 20: Table S3).

### Lentivirus infection to U2OS/2-6-3 cells and analysis of LacO-directed integration

Lentiviral solution (p24, 100 ng) was infected to 2.5 × 10^5^ U2OS/2-6-3 cells for 2 days. To quantitate the LacO-directed integration, gDNA was subjected to qPCR analysis with LacO-(sense or antisense)/Lenti-5237F, and TaqMan probe (pLenti6-LTR probe). For measuring the overall infectivity, cells were cultured in the presence of BlasticidineS (10 μg/ml) for 2 weeks. For normalizing the plating efficiency, colonies were formed in normal culture medium. Colonies were fixed with 70% ethanol and stained with Giemsa’s staining solution, and enumerated. Oligonucleotides used in this procedure are listed in (Additional file 20: Table S3).

### Cre mediated expression of Cherry-LacR-Vpr

U2OS/2-6-3+pCAL-loxP-CLV (263/loxP-CLV), in which expression of LacR-fused Vpr was switched on when Cre-recombinase was expressed, were first established by G418 selection. Then, cells were infected with adenovirus for LacZ (control) or Cre (inducing CLV expression) expression at multiplicity of infection (MOI) of 100 for 2 days, and subjected to live cell imaging and ChIP assay for Vpr (Additional file 8: Figure S7a, b). For siRNA experiments, 2.5 × 10^5^ cells were transfected with control or *Topo1* siRNA (50 nM) by Lipofectamine RNAiMax transfection reagent. After 1.5 days of siRNA transfection, adenovirus was infected for 2 days, and cells were subjected to following analysis; LacO-directed integration, native-ChIP assay for Topo1-cc and LM-PCR (Fig. 7g and Additional file 8: Figure S7c, d, respectively). Oligonucleotides used in this procedure are listed in (Additional file 20: Table S3).

### Linear amplification mediated-PCR

LAM-PCR was performed, as previously described [8] with some modification. Briefly, 1st liner PCR was performed using biotinylated-Lenti-5203F primer by two round of amplification with 50 cycles using Taq polymerase (Qiagen). After purification by SA-beads, dsDNA was synthesized with Klenow fragment (NEB) with random hexamer. Following extensive washes, dsDNA on SA-beads were digested with *Msp*I or *Nla*III, and ligated with corresponding dsDNA linker cassettes (LC). After that, 1st PCR was performed with biotinylated-M667/LC1, followed by SA-beads purification. And then, 2nd exponential PCR was performed with EV984/LC2. Size selection of amplified DNA was performed on agarose gel, and cloned into pGEM-T Easy vector. Sequencing was carried out by ABI3130x (Applied Biosystems), and integration sites were determined. Oligonucleotides used in this procedure are listed in (Additional file 20: Table S3).

### Quantitative reverse-transcription-PCR (qRT-PCR)

Total RNA was purified by RNeasy kit (Qiagen), and cDNA was generated by HighCapacity cDNA reverse transcription kit (Invitrogen). Quantitative-PCR was performed using SyBr premix ExTaq Tli RNaseH plus. The expression levels of each gene were normalized by the relative amounts of *β*-*Actin*. The primers used for qRT-PCR are listed in (Additional file 21: Table S4).

## Additional files

**Additional file 1: Figure S1.** Vpr induces structural alteration of DNA. **a** Representative AFM images of dsDNA in 1.0 μm × 1.0 μm field. **b** Representative raw data of Rq values of dsDNA. The Rq values of each dsDNA are shown as a plot, and the medians are indicated by red bars. **c** Relative Rq values of Vpr mutants. C-terminal mutants of Vpr were defective in DNA structural alteration. Data were obtained from more than three independent experiments. Error bar indicates ± SEM. In Fig. 1b, data of Buffer, Chlq, Wt, *Δ*C12 and Ct4RA are depicted.

**Additional file 2: Figure S2.** The interaction between Vpr and RPA70 (a) or T4gp32 (b) was not detected. **a** FLAG-Strep-Vpr (0.1, 0.317. 1.0 pmol) was incubated with rRPA70 (1.0 pmol), and pulled-down with Streptavidine M280 beads. Proteins were analysed by WB with indicated antibodies. **b** FLAG-Strep-Vpr (1, 3.17, 10 pmol) was incubated with T4gp32 (100 pmol), and pulled-down with Streptavidine M280 beads. Proteins were visualized by Oriole fluorescent gel staining.

**Additional file 3: Figure S3.** Vpr provokes Topo1 stress. **a** RADAR analysis detecting covalently bound DNA and Topo1. HEK293T cells were transfected with indicated Topo1-HA construct. Y723F is a catalytically inactive mutant of Topo1. A covalent complex of Topo1 and DNA was formed in the cells that were first transduced with Topo1-Wt, and then treated with CPT (20 μM, 1 h) or paraformaldehyde (PFA; 1 mM, 2 h), whereas the complex was only detected in cells with Topo1-Y723F when treated with PFA, but not with CPT. The same membrane was reprobed with α-dsDNA antibody after stripping. **b** Different amounts of DNA were blotted in the RADAR analysis.

**Additional file 4: Table S1.** Knockdown efficiency of siRNA target genes. Relative expression levels compared to cont-si (100%: shaded) are shown. Data were obtained from at least three independent experiments.

**Additional file 5: Figure S4.** Vpr-induced DNA damages are suppressed by TDP1 or RNaseH1 expression. **a** Exogenous expression of RNaseH1 (RNH1) and TDP1. Mit-23 cells were transfected with indicated vector (pFLAG-CMV2 based vector), and whole cell extracts were subjected to WB analysis. Arrowhead (black), TDP1; arrow, RNaseH1; arrowhead (red), Vpr; asterisk, non-specific bands. **b** RNH1 and TDP1 suppresses Vpr induced DDR. Mit-23 cells transfected with RNH1 or TDP1 showed reduced level of Vpr-induced phosphorylation of H2AX. A representative result out of two independent experiments is depicted.

**Additional file 6: Figure S5.** Forced accumulation of Vpr induced DDR in the vicinity of chromatin. **a–e** Forced accumulation of Vpr induces phosphorylation of H2AX at Ser139 (a), ATM at Ser1981 (b), RPA32 at Ser33 (c), Rad17 at Ser645 (d), and accumulation of mono- and poly-ubiquitin conjugates (e), on surrounding region.

**Additional file 7: Figure S6.** LM-PCR for detecting DSB-ends in the LacO repeats. **a**. Schematic of LM-PCR. Genomic DNA samples prepared from cells, which were transduced with LacR-construct were treated with T4 polymerase and T4 PNK for blunting the DSB ends, and ligated with blunt-end JW-linkers. LacO/JW-linker junctions were amplified by specific primers, shown in (b). **b**. Diagram of LacO/JW-linker amplification product. Arrows above the box indicate the PCR primers for JW-linker and LacO. **c** Sequencing chromatogram of LacO/JW-linker junction. Black arrow and red arrow indicate JW-linker and LacO repeats, respectively. **d** Representative amplification plot of LacO/JW-qPCR. Cherry-LacR fused-OVA, Black; -Fok1, blue; -Vpr, red curve. Gray curve show the standard samples with indicated copy numbers.

**Additional file 8: Figure S7.** *Topo1* is involved in Vpr-induced DDR and DSB. **a** Live cell imaging of CLV inducible cell line. To confirm effect of Vpr on Topo1-cc formation, we transfected pCAL-loxP-CLV to U2OS/2-6-3 cells, and obtained a cell line (263/loxP-CLV), in which expression of a Cherry-LacR fused Vpr (CLV) can be switched on after Cre expression. In the experiment, 263/loxP-CLV cells were infected with adenoviruses expressing LacZ (Ad-LacZ) or Cre (Ad-Cre) for 2 days at MOI of 100. Single focus of mCherry was distinctly observed in Ad-Cre infected cells (lower panel), suggesting that CLV was expressed in Cre-dependent manner. Scale bar indicates 20 μm. **b** Specific accumulation of CLV on the LacO repeats. ChIP assay with α-Vpr antibody (8D1) was performed in 263/loxP-CLV cells infected with Ad-LacZ or -Cre at 2dpi. **c** Down regulation of *Topo1* reduces Vpr induced Topo1-cc on the LacO repeats. After downregulation of *Topo1*, 263/loxP-CLV cells, which were infected with Ad-Cre, was subjected to native-ChIP assay with α-Topo1 antibody. A representative result out of two independent experiments is depicted. **d** Topo1 is required for Vpr-induced DSB on the LacO repeats. After downregulation of *Topo1*, 263/loxP-CLV cells were infected with Ad-LacZ or -Cre, and subjected to LM-PCR analysis to measure the extent of DSB on the LacO repeats. Vpr-induced DSB was calculated by subtracting the amounts of DSB observed in Ad-LacZ infected cells from those observed in Ad-Cre infected cells. Data were obtained from three independent experiments.

**Additional file 9: Figure S8.** Representative data of Lenti-LacO qPCR for detecting the LacO-directed integration. **a, b** Amplification plots of sense integration (a) and anti-sense integration (b), respectively. Blue (light blue ~ dark blue) and red (pink ~ red) curves show CV- and CLV-virus infected samples with different MOIs shown by amounts of p24, respectively. Gray curves indicate standard samples with indicated copy numbers. **c, d** Sequencing chromatograms of sense (c) and anti-sense (d) integration product. Blue arrows and red arrows indicate 3′-LTR and LacO repeats, respectively.

**Additional file 10: Figure S9.** Proviral integration in the vicinity of Vpr-induced DSB sites. Targeting of HIV-1 proviral DNA to CLV-induced DSB sites. U2OS/2-6-3 cells were first transfected with indicated construct, and then infected with NL-4-3/D64A/R− virus. The percentages of LacO directed integration per overall integration (*Alu*-*gag* two-step qPCR) are shown. Data were obtained from three independent experiments. Error bar indicates ± SEM. In Cherry-Vpr, Cherry-LacR-Vpr, and Fok1-Cherry-LacR, the *P*-value was 0.37, 0.051, and 0.015 for sense integration, respectively. The *P*-value for antisense-integration was 0.44, 0.001, and 5.21 × 10^−5^, respectively.

**Additional file 11: Figure S10.** Schematic of *Alu*-*gag* two-step nested qPCR. First PCR to amplify *Alu*-proviral DNA junction was performed using PCR primers targeting the *Alu* (black arrow) and *gag* (pink arrows). Red wavy line fused to *gag*-primer indicates the tag-sequence for 2nd qPCR primer binding. In second qPCR, viral DNA fragments were amplified by LTR primer (blue arrow) and tag-primer (red arrow). The green box indicates the position of the TaqMan probe for *gag*.

**Additional file 12: Figure S11.** Incorporation of Cherry-LacR-Vpr does not affect overall viral infectivity. U2OS/2-6-3 cells infected with indicated lentivirus, which had Blasticidine-resitance gene, were subjected to Blasticidine (Bsd) selection. The infected cells obtain Bsd resistance by the successful lentiviral integration. Data were obtained from three independent experiments. Error bar indicates ± SEM.

**Additional file 13: Figure S12.** A representative sequencing chromatogram of the LacO/proviral DNA junction, obtained by the LAM-PCR. Blue arrows and red arrows indicate 3′-LTR and LacO repeats, respectively.

**Additional file 14: Figure S13.** Topo1 is required for Vpr-dependent upregulation of viral infection. **a** MM-6 cells were infected with Vpr proficient (R+) or deficient (R−) NL4-3 viruses under down-regulation of *Topo1* by three species of siRNAs. The integration rate was quantitated by *Alu*-*gag* two-step nested qPCR at 2dpi; relative integration rates are shown. Data were obtained from more than three independent experiments. Error bar indicates ± SEM. **P < 0.05 **b** Knockdown efficiency of each siRNA. Relative levels of *Topo1* expression are shown. Topo1 siRNA#3 was used in other experiments.

**Additional file 15: Figure S14.** Vpr upregulates viral infection in differentiated MM-6 cells. **a** MM-6 cells were infected with Vpr proficient (R+) or deficient (R−) NL4-3-Luc/E- viruses. Luciferase assay was performed at 3dpi. Data were obtained from more than three independent experiments. Error bar indicates ± SEM. **b** MM-6 cells were infected with Vpr proficient (R+) or deficient (R−) NL4-3-EGFP/E- viruses. Percentage of EGFP positive cells was determined by FCM at 3dpi. Representative FCM data are shown in bottom panels. Green colored plots were gated as EGFP positive cells.

**Additional file 16: Figure S15.** XPG is required for Vpr-induced DDR and DSB. **a, b** Effects of downregulation of XPG on Vpr-induced DDR. The expression of *XPG* in Mit-23 cells was first downregulated by siRNA, and then Vpr expression was initiated on day 2 after introduction of *XPG* targeting siRNA. On day 1 after Vpr expression was started, phosphorylation of H2AX (a) and G_2_/M checkpoint activation (b) was analysed by flow cytometry. Downregulation of *XPG* significantly reduced Vpr-induced phosphorylation of H2AX (*P* = 4.4 × 10^−5^), and the G_2_/M arrest (*P* = 0.025). Data were obtained from three independent experiments. Error bar indicates ± SEM. **c** XPG is required for Vpr-induced DSBs. Neutral comet assay was performed using Mit-23 cell, the *XPG* expression of which was down-regulated. Data were obtained from three independent experiments. Error bar indicates ± SEM. *P* = 0.037. **d** XPG is required for rVpr-induced DSB in resting macrophages. Differentiated MM-6 cells were treated with 100 ng/ml of rVpr under the down-regulation of *XPG*, and subjected to neutral comet assay. Data were obtained from three independent experiments. Error bar indicates ± SEM. *P* = 0.001. **e** XPG is required for Vpr-induced upregulation of viral infection in resting macrophages. Differentiated MM-6 cells were infected with Vpr proficient (R+) or deficient (R−) NL4-3 viruses after transfection of siRNA, and the integration rate was quantitated by *Alu*-*gag* two-step nested qPCR; relative integration rates are shown. A representative result out of two independent experiments is depicted.

**Additional file 17: Figure S16.** Forced accumulation of Vpr increases formation of R-loops. **a** U2OS/2-6-3 cells were transfected with indicated LacR fused constructs. Two days after transfection, DNA samples were prepared and subjected to DRIP assay. DRIP assay was done with α-DNA/RNA hybrids antibody (S9.6). RNaseH treatments were performed to confirm the specificity of DRIP assay. Analysis of promotor (green column) and terminator (yellow column) regions of *Actin* gene were included in the qPCR analysis, as negative and positive control of DRIP assay, respectively. A representative result out of two independent experiments is depicted. **b** 263/loxP-CLV cells were infected with Ad-LacZ or -Cre. DNA samples were prepared and subjected to DRIP assay at 2dpi. Data were obtained from more than three independent experiments. Error bar indicates ± SEM.

**Additional file 18: Figure S17.** C-terminus of Vpr is required for topoisomer induction activity. DNA supercoiling assay was performed with increasing amounts of peptides (1, 10, 100 pmol; 0.015, 0.15, 1.5 Vpr molecules/bp). In the presence of C45, faster migrating topoisomers appeared.

**Additional file 19: Table S2.** List of resources used in this study.

**Additional file 20: Table S3.** List of oligonucleotides used in this study.

**Additional file 21: Table S4.** List of primers for qRT-PCR.

## Abbreviations

AFM: atomic force microscopy
ATR: ataxia-telangiectasia and Rad3-related
cART: combined antiretroviral therapy
ChIP: chromatin immunoprecipitation
CPT: camptothecin
DDR: DNA damage response
DSB: DNA double-strand break
FRAP: fluorescence recovery after photo-bleaching
HIV-1: human immunodeficiency virus-1
LM-PCR: ligation-mediated (LM)-PCR
MDMs: monocyte derived macrophages
MOI: multiplicity of infection
RADAR: rapid approach to DNA adducts recovery
RPA70: replication protein A 70-kDa subunit
SSB: DNA single-strand break
Topo1: topoisomerase 1
Topo1-cc: Topo1-covalent complex
Vpr: viral protein R

## References

[1] Trono D, Van Lint C, Rouzioux C, Verdin E, Barré-Sinoussi F, Chun TW (2010). HIV persistence and the prospect of long-term drug-free remissions for HIV-infected individuals. Science.

[2] Iglesias-Ussel MD, Romerio F (2011). HIV reservoirs: the new frontier. AIDS Rev.

[3] Abbas W, Tariq M, Iqbal M, Kumar A, Herbein G (2015). Eradication of HIV-1 from the macrophage reservoir: an uncertain goal?. Viruses.

[4] Cohen EA, Dehni G, Sodroski JG, Haseltine WA (1990). Human immunodeficiency virus vpr product is a virion-associated regulatory protein. J Virol.

[5] Balliet JW, Kolson DL, Eiger G, Kim FM, McGann KA, Srinivasan A (1994). Distinct effects in primary macrophages and lymphocytes of the human immunodeficiency virus type 1 accessory genes vpr, vpu, and nef: mutational analysis of a primary HIV-1 isolate. Virology.

[6] Connor RI, Chen BK, Choe S, Landau NR (1995). Vpr is required for efficient replication of human immunodeficiency virus type-1 in mononuclear phagocytes. Virology.

[7] Eckstein DA, Sherman MP, Penn ML, Chin PS, De Noronha CM, Greene WC (2001). HIV-1 Vpr enhances viral burden by facilitating infection of tissue macrophages but not nondividing CD4+ T cells. J Exp Med.

[8] Koyama T, Sun B, Tokunaga K, Tatsumi M, Ishizaka Y (2013). DNA damage enhances integration of HIV-1 into macrophages by overcoming integrase inhibition. Retrovirology.

[9] Guenzel CA, Hérate C, Benichou S (2014). HIV-1 Vpr-a still “enigmatic multitasker”. Front Microbiol.

[10] Roshal M, Kim B, Zhu Y, Nghiem P, Planelles V (2003). Activation of the ATR-mediated DNA damage response by the HIV-1 viral protein R. J Biol Chem.

[11] Zimmerman ES, Chen J, Andersen JL, Ardon O, Dehart JL, Blackett J (2004). Human immunodeficiency virus type 1 Vpr-mediated G2 arrest requires Rad17 and Hus1 and induces nuclear BRCA1 and gamma-H2AX focus formation. Mol Cell Biol.

[12] Lai M, Zimmerman ES, Planelles V, Chen J (2005). Activation of the ATR pathway by human immunodeficiency virus type 1 Vpr involves its direct binding to chromatin in vivo. J Virol.

[13] Nakai-Murakami C, Shimura M, Kinomoto M, Takizawa Y, Tokunaga K, Taguchi T (2007). HIV-1 Vpr induces ATM-dependent cellular signal with enhanced homologous recombination. Oncogene.

[14] Laguette N, Brégnard C, Hue P, Basbous J, Yatim A, Larroque M (2014). Premature activation of the SLX4 complex by Vpr promotes G2/M arrest and escape from innate immune sensing. Cell.

[15] Morellet N, Bouaziz S, Petitjean P, Roques BP (2003). NMR structure of the HIV-1 regulatory protein VPR. J Mol Biol.

[16] Tachiwana H, Shimura M, Nakai-Murakami C, Tokunaga K, Takizawa Y, Sata T (2006). HIV-1 Vpr induces DNA double-strand breaks. Cancer Res.

[17] Shimura M, Tanaka Y, Nakamura S, Minemoto Y, Yamashita K, Hatake K (1999). Micronuclei formation and aneuploidy induced by Vpr, an accessory gene of human immunodeficiency virus type 1. FASEB J.

[18] Groschel B, Bushman F (2005). Cell cycle arrest in G2/M promotes early steps of infection by human immunodeficiency virus. J Virol.

[19] Zimmerman ES, Sherman MP, Blackett JL, Neidleman JA, Kreis C, Mundt P (2006). Human immunodeficiency virus type 1 Vpr induces DNA replication stress in vitro and in vivo. J Virol.

[20] Zou L, Elledge SJ (2003). Sensing DNA damage through ATRIP recognition of RPA-ssDNA complexes. Science.

[21] DeHart JL, Zimmerman ES, Ardon O, Monteiro-Filho CM, Argañaraz ER, Planelles V (2007). HIV-1 Vpr activates the G2 checkpoint through manipulation of the ubiquitin proteasome system. Virol J.

[22] Belzile JP, Duisit G, Rougeau N, Mercier J, Finzi A, Cohen EA (2007). HIV-1 Vpr-mediated G2 arrest involves the DDB1-CUL4AVPRBP E3 ubiquitin ligase. PLoS Pathog.

[23] Belzile JP, Richard J, Rougeau N, Xiao Y, Cohen EA (2010). HIV-1 Vpr induces the K48-linked polyubiquitination and proteasomal degradation of target cellular proteins to activate ATR and promote G2 arrest. J Virol.

[24] Tan L, Ehrlich E, Yu XF (2007). DDB1 and Cul4A are required for human immunodeficiency virus type 1 Vpr-induced G2 arrest. J Virol.

[25] Kichler A, Pages JC, Leborgne C, Druillennec S, Lenoir C, Coulaud D (2000). Efficient DNA transfection mediated by the C-terminal domain of human immunodeficiency virus type 1 viral protein R. J Virol.

[26] Lyonnais S, Gorelick RJ, Heniche-Boukhalfa F, Bouaziz S, Parissi V, Mouscadet JF (2013). A protein ballet around the viral genome orchestrated by HIV-1 reverse transcriptase leads to an architectural switch: from nucleocapsid-condensed RNA to Vpr-bridged DNA. Virus Res.

[27] Pommier Y, Sun Y, Huang SN, Nitiss JL (2016). Roles of eukaryotic topoisomerases in transcription, replication and genomic stability. Nat Rev Mol Cell Biol.

[28] Sordet O, Redon CE, Guirouilh-Barbat J, Smith S, Solier S, Douarre C (2009). Ataxia telangiectasia mutated activation by transcription- and topoisomerase I-induced DNA double-strand breaks. EMBO Rep.

[29] Eldin P, Chazal N, Fenard D, Bernard E, Guichou JF, Briant L (2014). Vpr expression abolishes the capacity of HIV-1 infected cells to repair uracilated DNA. Nucleic Acids Res.

[30] Lahouassa H, Blondot ML, Chauveau L, Chougui G, Morel M, Leduc M (2016). HIV-1 Vpr degrades the HLTF DNA translocase in T cells and macrophages. Proc Natl Acad Sci USA.

[31] Murphy PJ, Shannon M, Goertz J (2011). Visualization of recombinant DNA and protein complexes using atomic force microscopy. J Vis Exp.

[32] Mizoguchi I, Ooe Y, Hoshino S, Shimura M, Kasahara T, Kano S (2005). Improved gene expression in resting macrophages using an oligopeptide derived from Vpr of human immunodeficiency virus type-1. Biochem Biophys Res Commun.

[33] Shamoo Y, Friedman AM, Parsons MR, Konigsberg WH, Steitz TA (1995). Crystal structure of a replication fork single-stranded DNA binding protein (T4 gp32) complexed to DNA. Nature.

[34] Jose D, Weitzel SE, Baase WA, von Hippel PH (2015). Mapping the interactions of the single-stranded DNA binding protein of bacteriophage T4 (gp32) with DNA lattices at single nucleotide resolution: gp32 monomer binding. Nucleic Acids Res.

[35] Nitiss JL, Soans E, Rogojina A, Seth A, Mishina M (2012). Topoisomerase assays. Curr Protoc Pharmacol.

[36] Desai SD, Zhang H, Rodriguez-Bauman A, Yang JM, Wu X, Gounder MK (2003). Transcription-dependent degradation of topoisomerase I-DNA covalent complexes. Mol Cell Biol.

[37] Steinacher R, Osman F, Lorenz A, Bryer C, Whitby MC (2013). Slx8 removes Pli1-dependent protein-SUMO conjugates including SUMOylated topoisomerase I to promote genome stability. PLoS ONE.

[38] Shimura M, Toyoda Y, Iijima K, Kinomoto M, Tokunaga K, Yoda K (2011). Epigenetic displacement of HP1 from heterochromatin by HIV-1 Vpr causes premature sister chromatid separation. J Cell Biol.

[39] Kiianitsa K, Maizels N (2013). A rapid and sensitive assay for DNA-protein covalent complexes in living cells. Nucleic Acids Res.

[40] Janicki SM, Tsukamoto T, Salghetti SE, Tansey WP, Sachidanandam R, Prasanth KV (2004). From silencing to gene expression: real-time analysis in single cells. Cell.

[41] Soutoglou E, Dorn JF, Sengupta K, Jasin M, Nussenzweig A, Ried T, Danuser G, Misteli T (2007). Positional stability of single double-strand breaks in mammalian cells. Nat Cell Biol.

[42] Soutoglou E, Misteli T (2008). Activation of the cellular DNA damage response in the absence of DNA lesions. Science.

[43] Naughton C, Avlonitis N, Corless S, Prendergast JG, Mati IK, Eijk PP (2013). Transcription forms and remodels supercoiling domains unfolding large-scale chromatin structures. Nat Struct Mol Biol.

[44] Anders L, Guenther MG, Qi J, Fan ZP, Marineau JJ, Rahl PB (2014). Genome-wide localization of small molecules. Nat Biotechnol.

[45] Kimura H, Cook PR (2001). Kinetics of core histones in living human cells: little exchange of H3 and H4 and some rapid exchange of H2B. J Cell Biol.

[46] Tóth KF, Knoch TA, Wachsmuth M, Frank-Stöhr M, Stöhr M, Bacher CP (2004). Trichostatin A-induced histone acetylation causes decondensation of interphase chromatin. J Cell Sci.

[47] Daniel R, Ramcharan J, Rogakou E, Taganov KD, Greger JG, Bonner W (2004). Histone H2AX is phosphorylated at sites of retroviral DNA integration but is dispensable for postintegration repair. J Biol Chem.

[48] Cooper A, García M, Petrovas C, Yamamoto T, Koup RA, Nabel GJ (2013). HIV-1 causes CD4 cell death through DNA-dependent protein kinase during viral integration. Nature.

[49] Beard P, Hughes M, Nyfeler K, Hoey M (1984). Unwinding of the DNA helix in simian virus 40 chromosome templates by RNA polymerase. Eur J Biochem.

[50] Sekulic N, Bassett EA, Rogers DJ, Black BE (2010). The structure of (CENP-A-H4)_2_ reveals physical features that mark centromeres. Nature.

[51] Pavri R, Zhu B, Li G, Trojer P, Mandal S, Shilatifard A (2006). Histone H2B monoubiquitination functions cooperatively with FACT to regulate elongation by RNA polymerase II. Cell.

[52] Nakamura K, Kato A, Kobayashi J, Yanagihara H, Sakamoto S, Oliveira DV (2011). Regulation of homologous recombination by RNF20-dependent H2B ubiquitination. Mol Cell.

[53] Zeng M, Ren L, Mizuno K, Nestoras K, Wang H, Tang Z (2016). CRL4(Wdr70) regulates H2B monoubiquitination and facilitates Exo1-dependent resection. Nat Commun.

[54] Taneichi D, Iijima K, Doi A, Koyama T, Minemoto Y, Tokunaga K (2011). Identification of SNF2 h, a chromatin-remodeling factor, as a novel binding protein of Vpr of human immunodeficiency virus type 1. J Neuroimmune Pharmacol.

[55] Maudet C, Sourisce A, Dragin L, Lahouassa H, Rain JC, Bouaziz S (2013). HIV-1 Vpr induces the degradation of ZIP and sZIP, adaptors of the NuRD chromatin remodeling complex, by hijacking DCAF1/VprBP. PLoS ONE.

[56] Romani B, Baygloo NS, Hamidi-Fard M, Aghasadeghi MR, Allahbakhshi E (2016). HIV-1 Vpr protein induces proteasomal degradation of chromatin-associated class I HDACs to overcome latent infection of macrophages. J Biol Chem.

[57] Deng C, Brown JA, You D, Brown JM (2005). Multiple endonucleases function to repair covalent topoisomerase I complexes in Saccharomyces cerevisiae. Genetics.

[58] Kim Y, Spitz GS, Veturi U, Lach FP, Auerbach AD, Smogorzewska A (2013). Regulation of multiple DNA repair pathways by the Fanconi anemia protein SLX4. Blood.

[59] Sollier J, Stork CT, García-Rubio ML, Paulsen RD, Aguilera A, Cimprich KA (2014). Transcription-coupled nucleotide excision repair factors promote R-loop-induced genome instability. Mol Cell.

[60] Sollier J, Cimprich KA (2015). Breaking bad: R-loops and genome integrity. Trends Cell Biol.

[61] Fregoso OI, Emerman M (2016). Activation of the DNA damage response is a conserved function of HIV-1 and HIV-2 Vpr that is independent of SLX4 recruitment. MBio.

[62] Smith JA, Daniel R (2011). Up-regulation of HIV-1 transduction in nondividing cells by double-strand DNA break-inducing agents. Biotechnol Lett.

[63] Ebina H, Kanemura Y, Suzuki Y, Urata K, Misawa N, Koyanagi Y (2012). Integrase-independent HIV-1 infection is augmented under conditions of DNA damage and produces a viral reservoir. Virology.

[64] Siddiqui K, Del Valle L, Morellet N, Cui J, Ghafouri M, Mukerjee R (2008). Molecular mimicry in inducing DNA damage between HIV-1 Vpr and the anticancer agent, cisplatin. Oncogene.

[65] Levy DN, Refaeli Y, MacGregor RR, Weiner DB (1994). Serum Vpr regulates productive infection and latency of human immunodeficiency virus type 1. Proc Natl Acad Sci USA.

[66] Power C, Hui E, Vivithanaporn P, Acharjee S, Polyak M (2012). Delineating HIV-associated neurocognitive disorders using transgenic models: the neuropathogenic actions of Vpr. J Neuroimmune Pharmacol.

[67] Katyal S, Lee Y, Nitiss KC, Downing SM, Li Y, Shimada M (2014). Aberrant topoisomerase-1 DNA lesions are pathogenic in neurodegenerative genome instability syndromes. Nat Neurosci.

[68] Saylor D, Dickens AM, Sacktor N, Haughey N, Slusher B, Pletnikov M (2016). HIV-associated neurocognitive disorder—pathogenesis and prospects for treatment. Nat Rev Neurol.

[69] Skourti-Stathaki K, Kamieniarz-Gdula K, Proudfoot NJ (2014). R-loops induce repressive chromatin marks over mammalian gene terminators. Nature.

