# Supplementary figures and images for: Structural alteration of DNA induced by viral protein R of HIV-1 triggers the DNA damage response
Source: Retrovirology. 2018 Jan 16;15:8. doi: 10.1186/s12977-018-0391-8 (PMC5771197; doi:10.1186/s12977-018-0391-8)

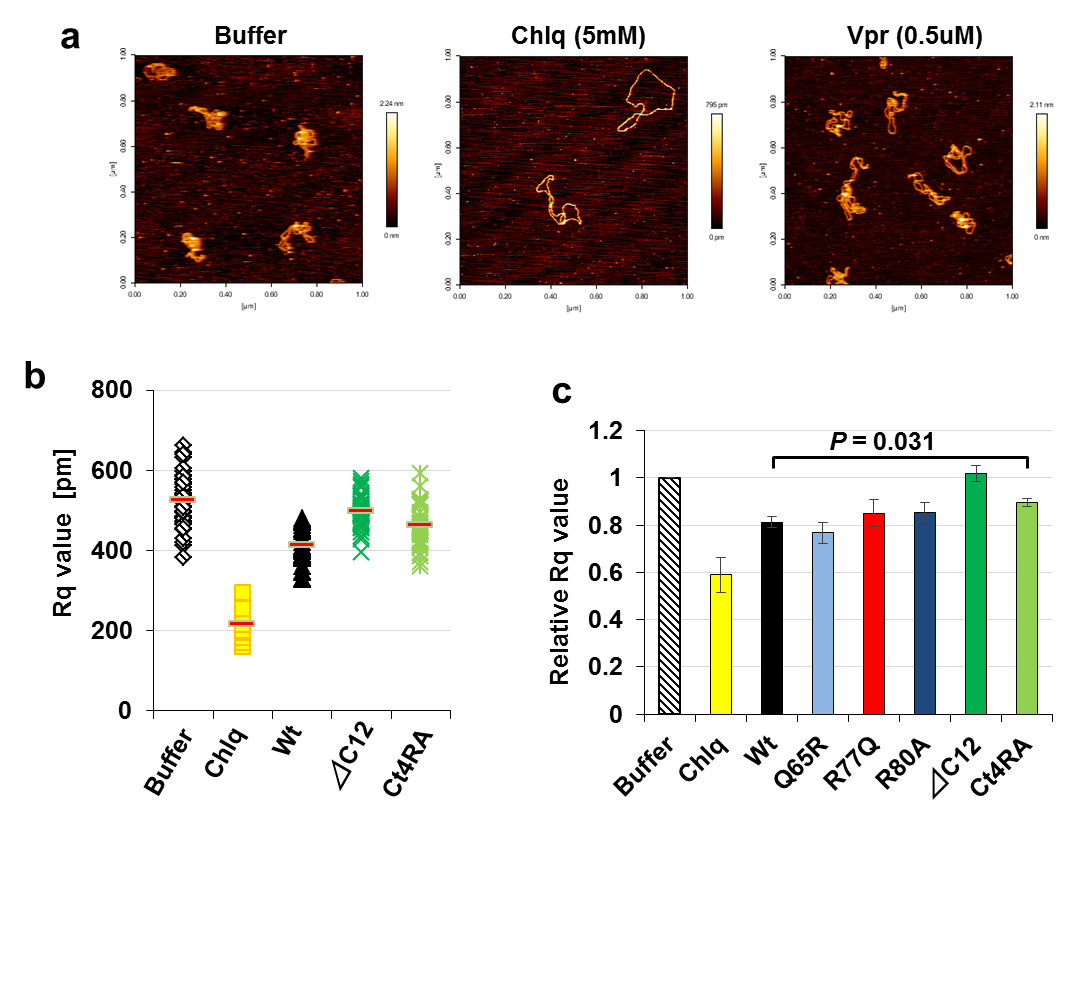

Supplement: Supplementary file 1 — Additional file 1: Figure S1. Vpr induces structural alteration of DNA. a Representative AFM images of dsDNA in 1.0 μm × 1.0 μm field. b Representative raw data of Rq values of dsDNA. The Rq values of each dsDNA are shown as a plot, and the medians are indicated by red bars. c Relative Rq values of Vpr mutants. C-terminal mutants of Vpr were defective in DNA structural alteration. Data were obtained from more than three independent experiments. Error bar indicates ± SEM. In Fig. 1b, data of Buffer, Chlq, Wt, ΔC12 and Ct4RA are depicted. [file 12977_2018_391_MOESM1_ESM.tif]

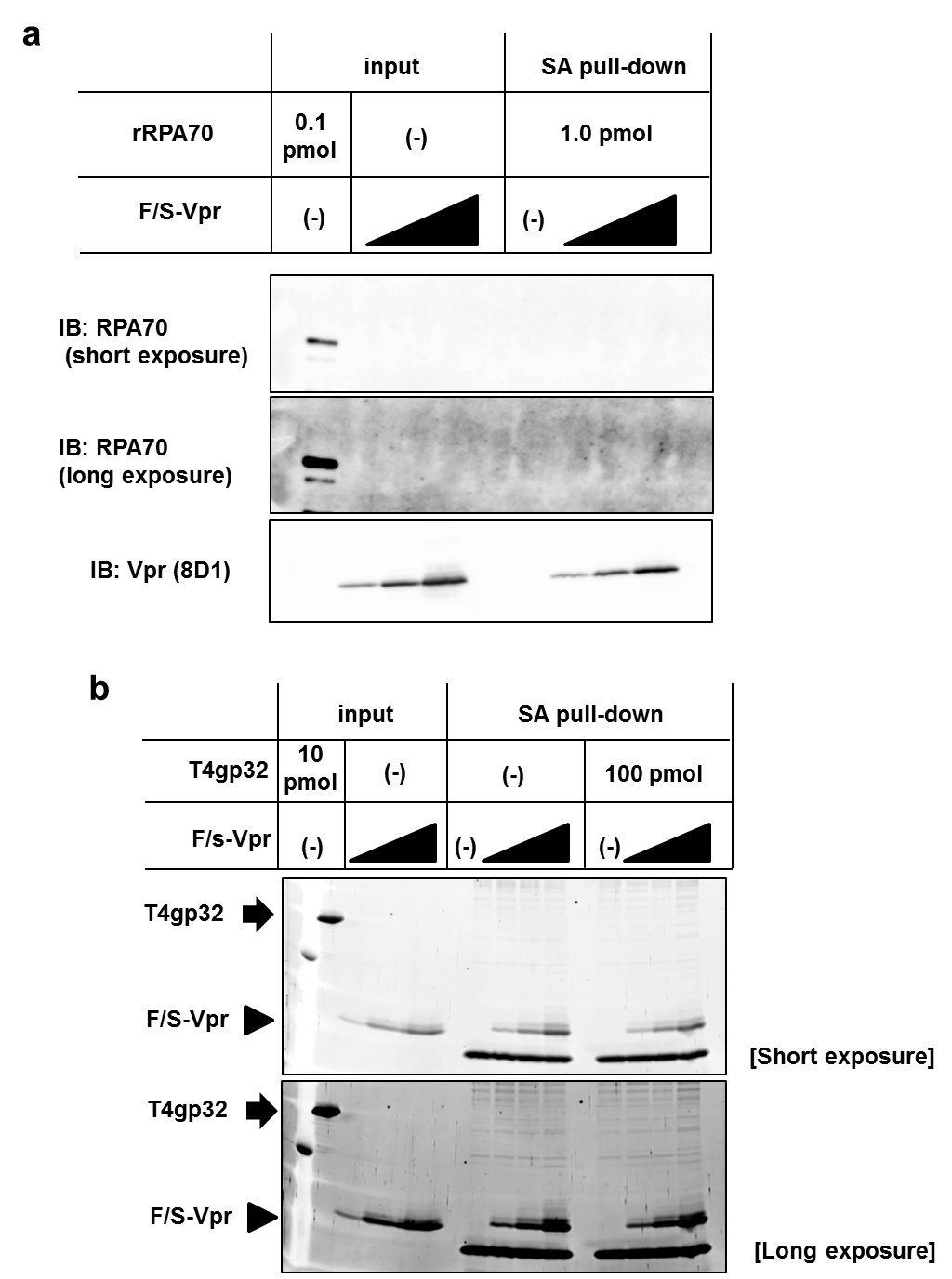

Supplement: Supplementary file 2 — Additional file 2: Figure S2. The interaction between Vpr and RPA70 (a) or T4gp32 (b) was not detected. a FLAG-Strep-Vpr (0.1, 0.317. 1.0 pmol) was incubated with rRPA70 (1.0 pmol), and pulled-down with Streptavidine M280 beads. Proteins were analysed by WB with indicated antibodies. b FLAG-Strep-Vpr (1, 3.17, 10 pmol) was incubated with T4gp32 (100 pmol), and pulled-down with Streptavidine M280 beads. Proteins were visualized by Oriole fluorescent gel staining. [file 12977_2018_391_MOESM2_ESM.tif]

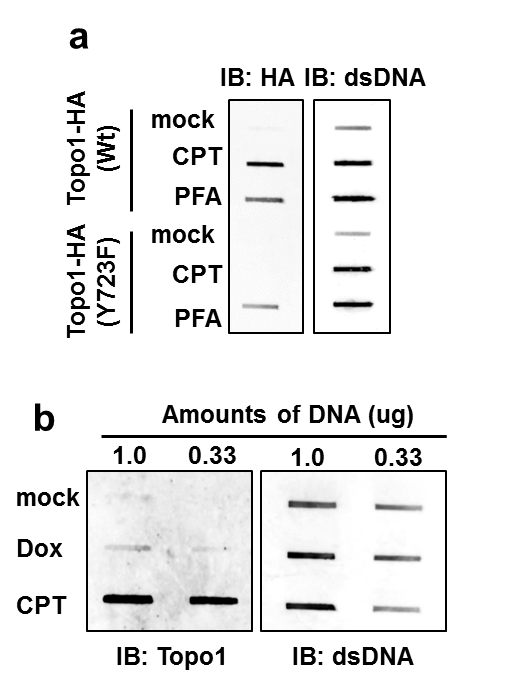

Supplement: Supplementary file 3 — Additional file 3: Figure S3. Vpr provokes Topo1 stress. a RADAR analysis detecting covalently bound DNA and Topo1. HEK293T cells were transfected with indicated Topo1-HA construct. Y723F is a catalytically inactive mutant of Topo1. A covalent complex of Topo1 and DNA was formed in the cells that were first transduced with Topo1-Wt, and then treated with CPT (20 μM, 1 h) or paraformaldehyde (PFA; 1 mM, 2 h), whereas the complex was only detected in cells with Topo1-Y723F when treated with PFA, but not with CPT. The same membrane was reprobed with α-dsDNA antibody after stripping. b Different amounts of DNA were blotted in the RADAR analysis. [file 12977_2018_391_MOESM3_ESM.tif]

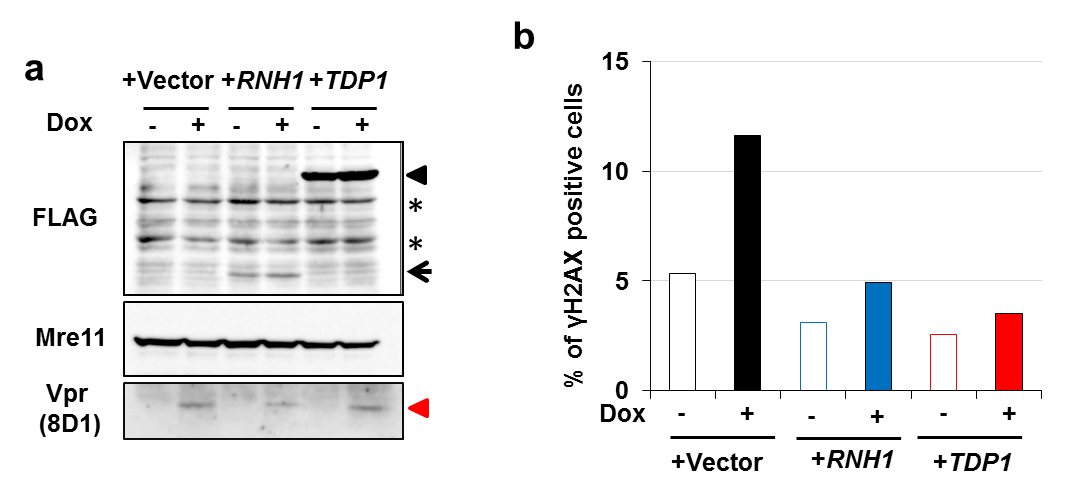

Supplement: Supplementary file 5 — Additional file 5: Figure S4. Vpr-induced DNA damages are suppressed by TDP1 or RNaseH1 expression. a Exogenous expression of RNaseH1 (RNH1) and TDP1. Mit-23 cells were transfected with indicated vector (pFLAG-CMV2 based vector), and whole cell extracts were subjected to WB analysis. Arrowhead (black), TDP1; arrow, RNaseH1; arrowhead (red), Vpr; asterisk, non-specific bands. b RNH1 and TDP1 suppresses Vpr induced DDR. Mit-23 cells transfected with RNH1 or TDP1 showed reduced level of Vpr-induced phosphorylation of H2AX. A representative result out of two independent experiments is depicted. [file 12977_2018_391_MOESM5_ESM.tif]

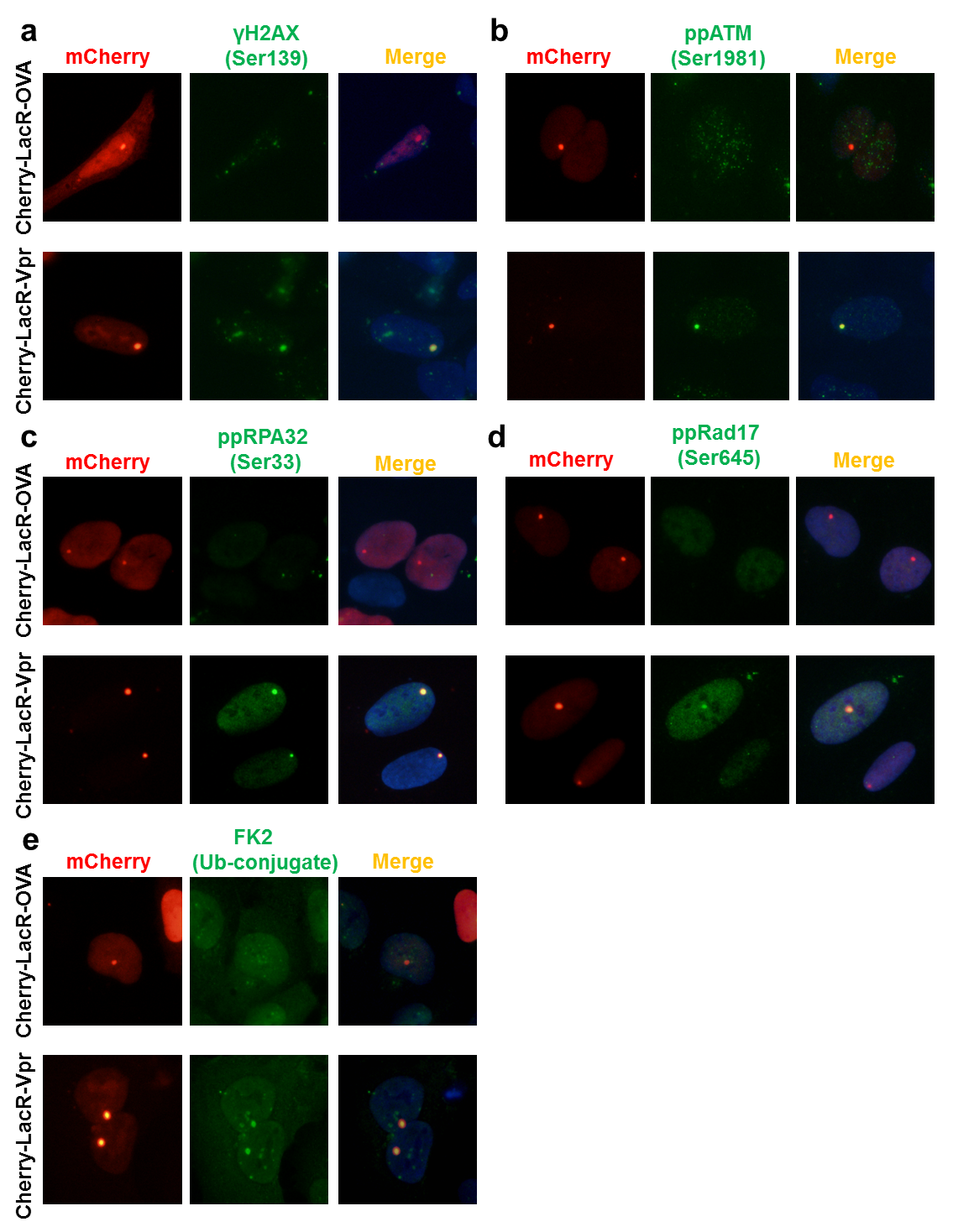

Supplement: Supplementary file 6 — Additional file 6: Figure S5. Forced accumulation of Vpr induced DDR in the vicinity of chromatin. a–e Forced accumulation of Vpr induces phosphorylation of H2AX at Ser139 (a), ATM at Ser1981 (b), RPA32 at Ser33 (c), Rad17 at Ser645 (d), and accumulation of mono- and poly-ubiquitin conjugates (e), on surrounding region. [file 12977_2018_391_MOESM6_ESM.tif]

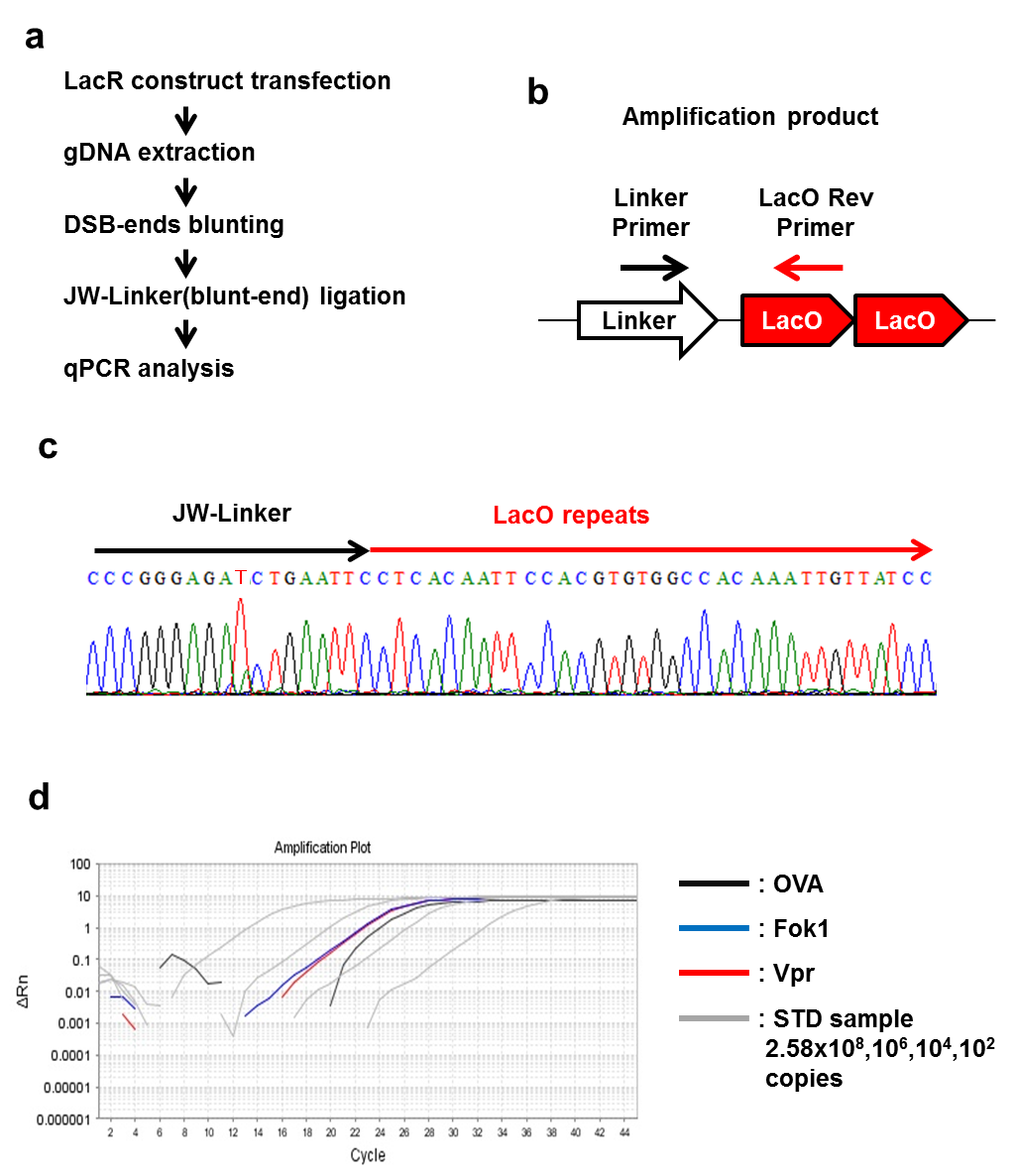

Supplement: Supplementary file 7 — Additional file 7: Figure S6. LM-PCR for detecting DSB-ends in the LacO repeats. a. Schematic of LM-PCR. Genomic DNA samples prepared from cells, which were transduced with LacR-construct were treated with T4 polymerase and T4 PNK for blunting the DSB ends, and ligated with blunt-end JW-linkers. LacO/JW-linker junctions were amplified by specific primers, shown in (b). b. Diagram of LacO/JW-linker amplification product. Arrows above the box indicate the PCR primers for JW-linker and LacO. c Sequencing chromatogram of LacO/JW-linker junction. Black arrow and red arrow indicate JW-linker and LacO repeats, respectively. d Representative amplification plot of LacO/JW-qPCR. Cherry-LacR fused-OVA, Black; -Fok1, blue; -Vpr, red curve. Gray curve show the standard samples with indicated copy numbers. [file 12977_2018_391_MOESM7_ESM.tif]

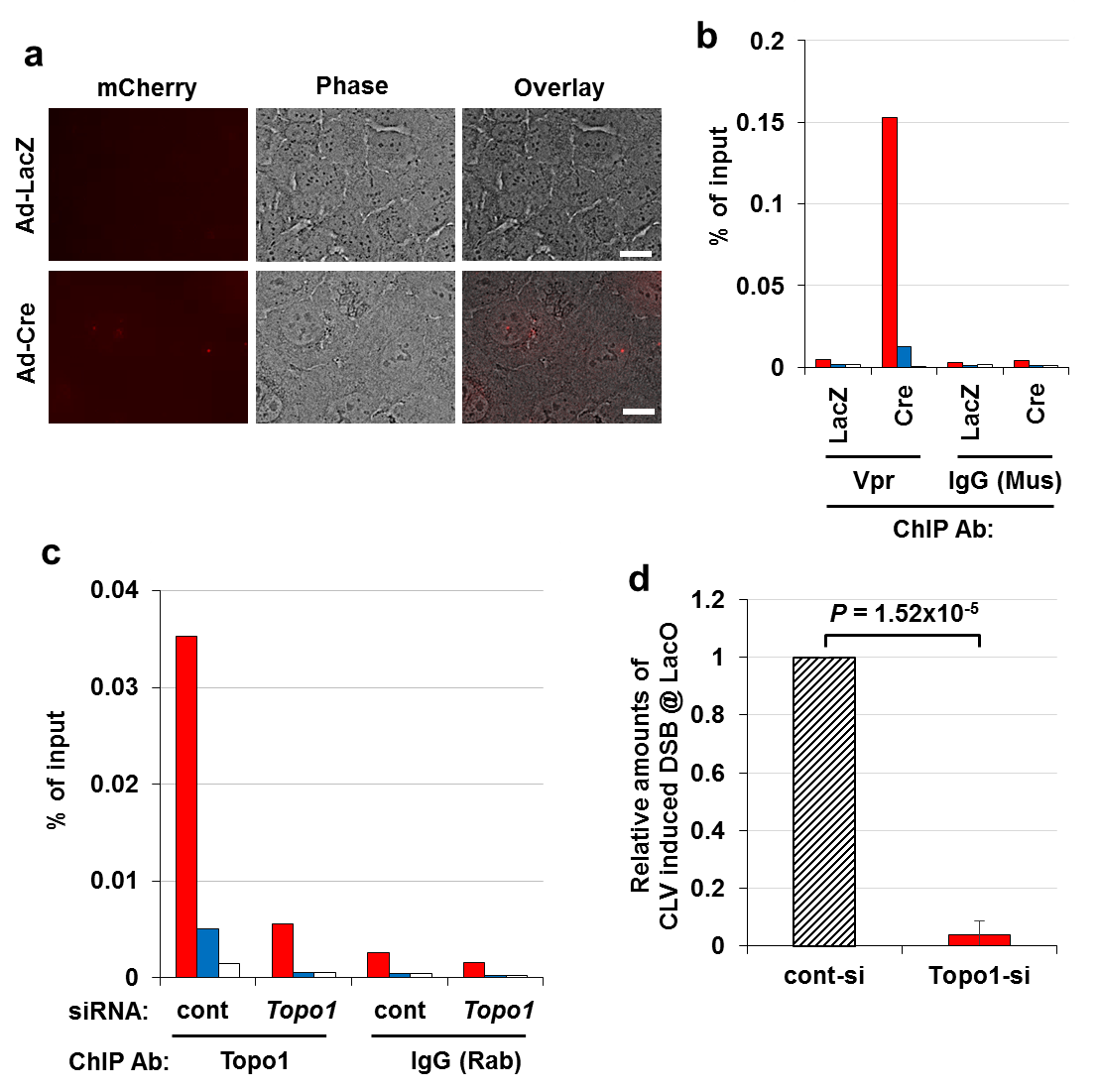

Supplement: Supplementary file 8 — Additional file 8: Figure S7. Topo1 is involved in Vpr-induced DDR and DSB. a Live cell imaging of CLV inducible cell line. To confirm effect of Vpr on Topo1-cc formation, we transfected pCAL-loxP-CLV to U2OS/2-6-3 cells, and obtained a cell line (263/loxP-CLV), in which expression of a Cherry-LacR fused Vpr (CLV) can be switched on after Cre expression. In the experiment, 263/loxP-CLV cells were infected with adenoviruses expressing LacZ (Ad-LacZ) or Cre (Ad-Cre) for 2 days at MOI of 100. Single focus of mCherry was distinctly observed in Ad-Cre infected cells (lower panel), suggesting that CLV was expressed in Cre-dependent manner. Scale bar indicates 20 μm. b Specific accumulation of CLV on the LacO repeats. ChIP assay with α-Vpr antibody (8D1) was performed in 263/loxP-CLV cells infected with Ad-LacZ or -Cre at 2dpi. c Down regulation of Topo1 reduces Vpr induced Topo1-cc on the LacO repeats. After downregulation of Topo1, 263/loxP-CLV cells, which were infected with Ad-Cre, was subjected to native-ChIP assay with α-Topo1 antibody. A representative result out of two independent experiments is depicted. d Topo1 is required for Vpr-induced DSB on the LacO repeats. After downregulation of Topo1, 263/loxP-CLV cells were infected with Ad-LacZ or -Cre, and subjected to LM-PCR analysis to measure the extent of DSB on the LacO repeats. Vpr-induced DSB was calculated by subtracting the amounts of DSB observed in Ad-LacZ infected cells from those observed in Ad-Cre infected cells. Data were obtained from three independent experiments. [file 12977_2018_391_MOESM8_ESM.tif]

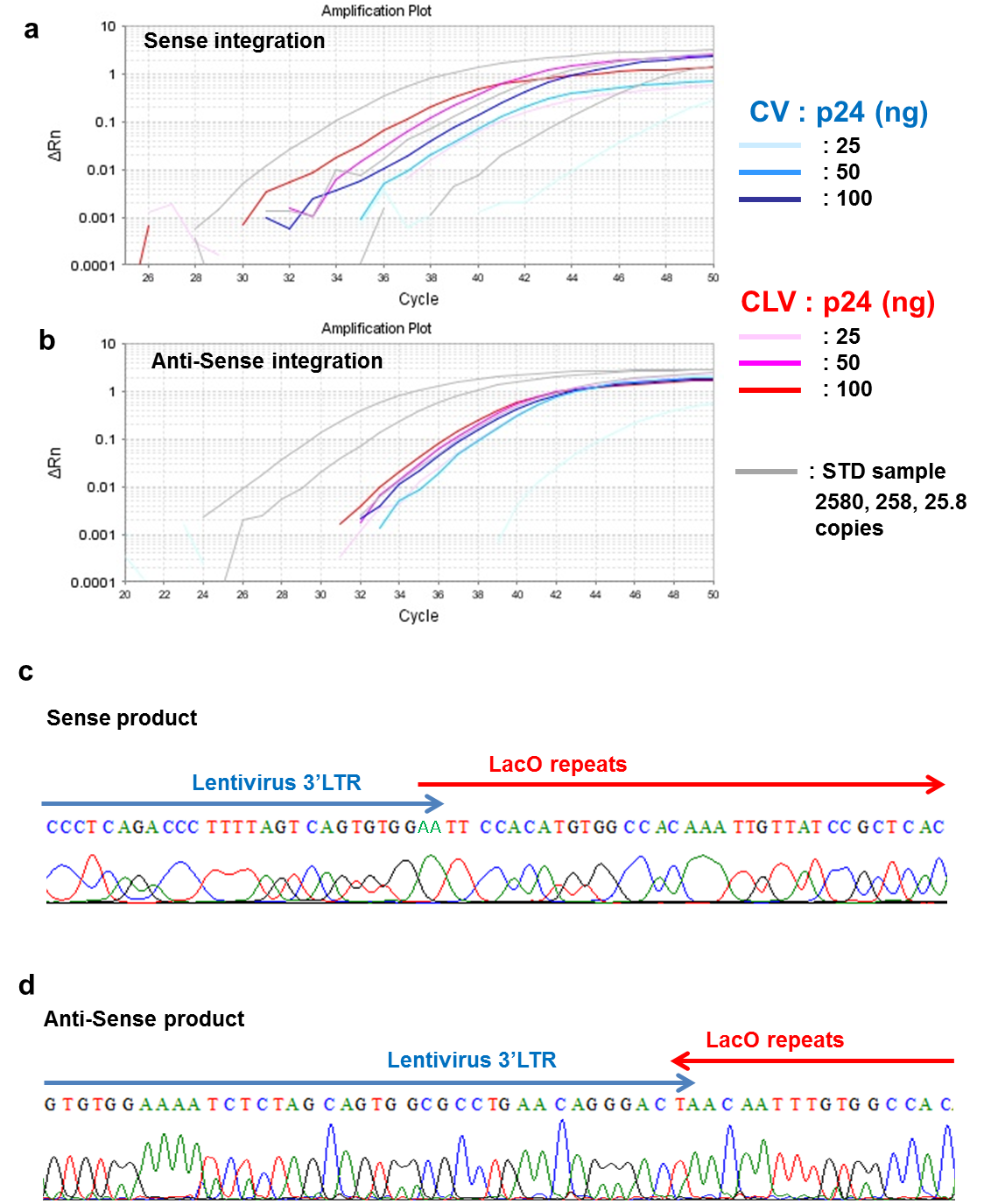

Supplement: Supplementary file 9 — Additional file 9: Figure S8. Representative data of Lenti-LacO qPCR for detecting the LacO-directed integration. a, b Amplification plots of sense integration (a) and anti-sense integration (b), respectively. Blue (light blue ~ dark blue) and red (pink ~ red) curves show CV- and CLV-virus infected samples with different MOIs shown by amounts of p24, respectively. Gray curves indicate standard samples with indicated copy numbers. c, d Sequencing chromatograms of sense (c) and anti-sense (d) integration product. Blue arrows and red arrows indicate 3′-LTR and LacO repeats, respectively. [file 12977_2018_391_MOESM9_ESM.tif]

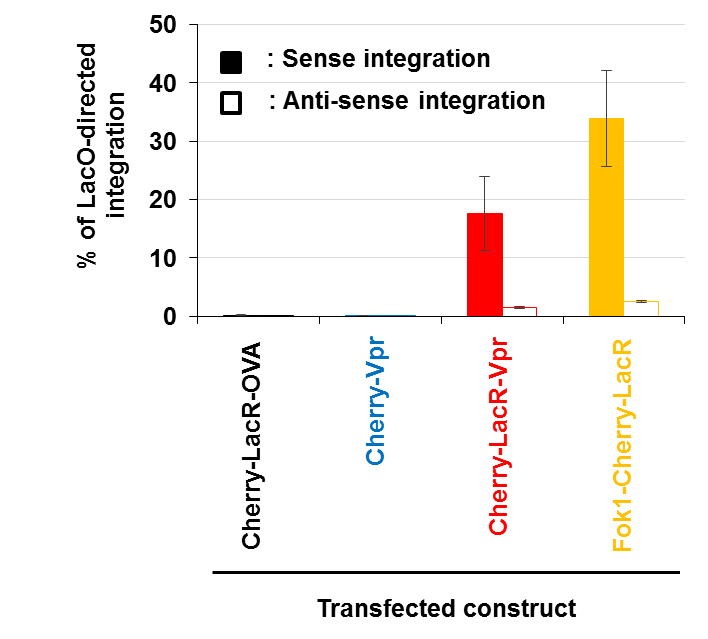

Supplement: Supplementary file 10 — Additional file 10: Figure S9. Proviral integration in the vicinity of Vpr-induced DSB sites. Targeting of HIV-1 proviral DNA to CLV-induced DSB sites. U2OS/2-6-3 cells were first transfected with indicated construct, and then infected with NL-4-3/D64A/R− virus. The percentages of LacO directed integration per overall integration (Alu-gag two-step qPCR) are shown. Data were obtained from three independent experiments. Error bar indicates ± SEM. In Cherry-Vpr, Cherry-LacR-Vpr, and Fok1-Cherry-LacR, the P-value was 0.37, 0.051, and 0.015 for sense integration, respectively. The P-value for antisense-integration was 0.44, 0.001, and 5.21 × 10^−5^, respectively. [file 12977_2018_391_MOESM10_ESM.tif]

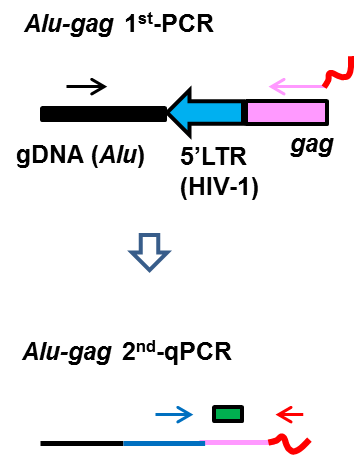

Supplement: Supplementary file 11 — Additional file 11: Figure S10. Schematic of Alu-gag two-step nested qPCR. First PCR to amplify Alu-proviral DNA junction was performed using PCR primers targeting the Alu (black arrow) and gag (pink arrows). Red wavy line fused to gag-primer indicates the tag-sequence for 2nd qPCR primer binding. In second qPCR, viral DNA fragments were amplified by LTR primer (blue arrow) and tag-primer (red arrow). The green box indicates the position of the TaqMan probe for gag. [file 12977_2018_391_MOESM11_ESM.tif]

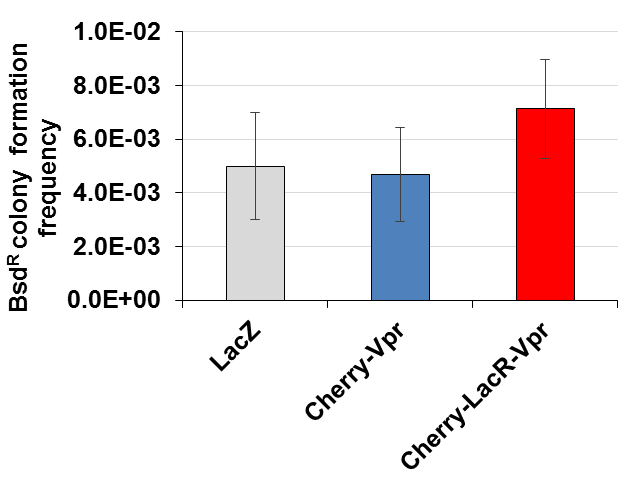

Supplement: Supplementary file 12 — Additional file 12: Figure S11. Incorporation of Cherry-LacR-Vpr does not affect overall viral infectivity. U2OS/2-6-3 cells infected with indicated lentivirus, which had Blasticidine-resitance gene, were subjected to Blasticidine (Bsd) selection. The infected cells obtain Bsd resistance by the successful lentiviral integration. Data were obtained from three independent experiments. Error bar indicates ± SEM. [file 12977_2018_391_MOESM12_ESM.tif]

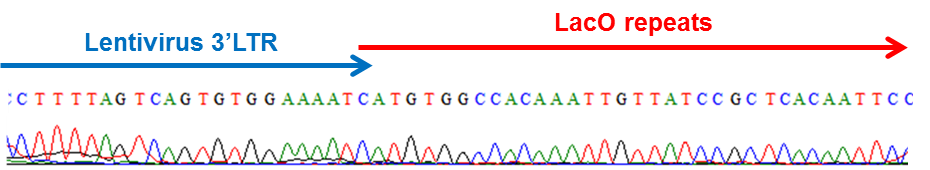

Supplement: Supplementary file 13 — Additional file 13: Figure S12. A representative sequencing chromatogram of the LacO/proviral DNA junction, obtained by the LAM-PCR. Blue arrows and red arrows indicate 3′-LTR and LacO repeats, respectively. [file 12977_2018_391_MOESM13_ESM.tif]

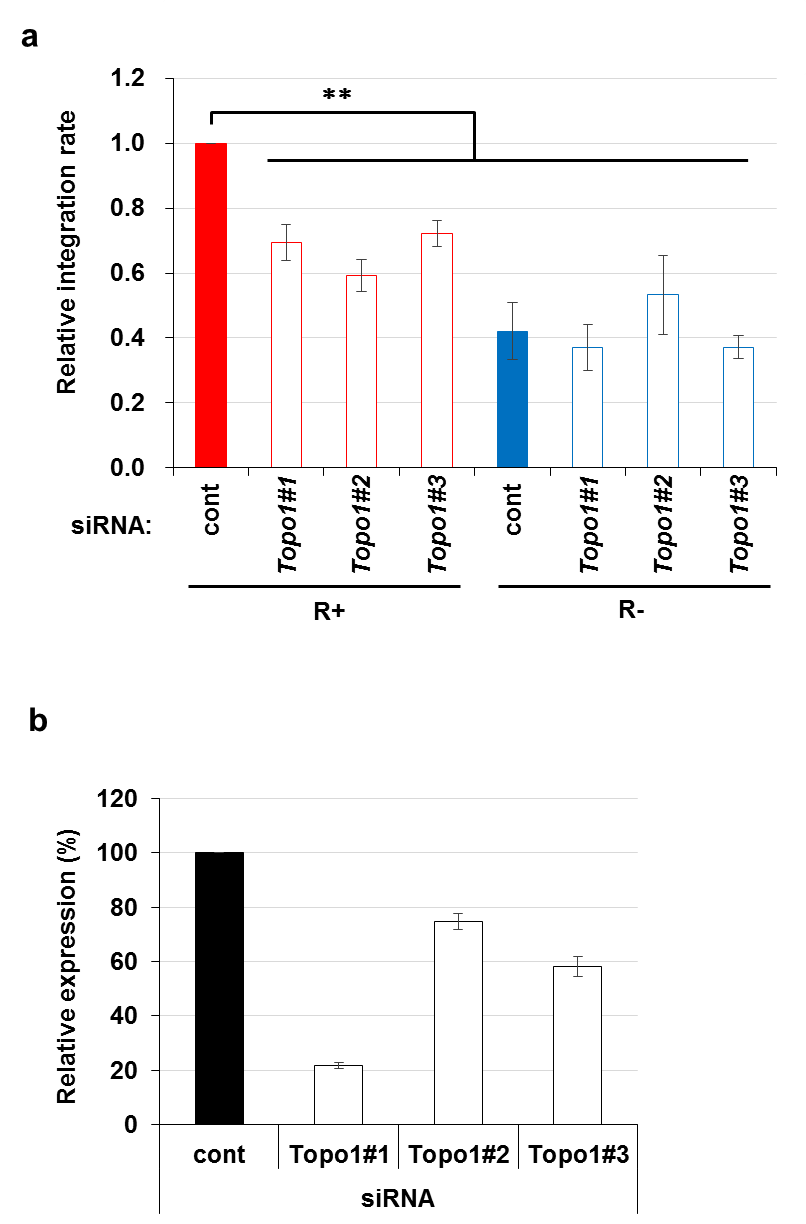

Supplement: Supplementary file 14 — Additional file 14: Figure S13. Topo1 is required for Vpr-dependent upregulation of viral infection. a MM-6 cells were infected with Vpr proficient (R+) or deficient (R−) NL4-3 viruses under down-regulation of Topo1 by three species of siRNAs. The integration rate was quantitated by Alu-gag two-step nested qPCR at 2dpi; relative integration rates are shown. Data were obtained from more than three independent experiments. Error bar indicates ± SEM. **P < 0.05 b Knockdown efficiency of each siRNA. Relative levels of Topo1 expression are shown. Topo1 siRNA#3 was used in other experiments. [file 12977_2018_391_MOESM14_ESM.tif]

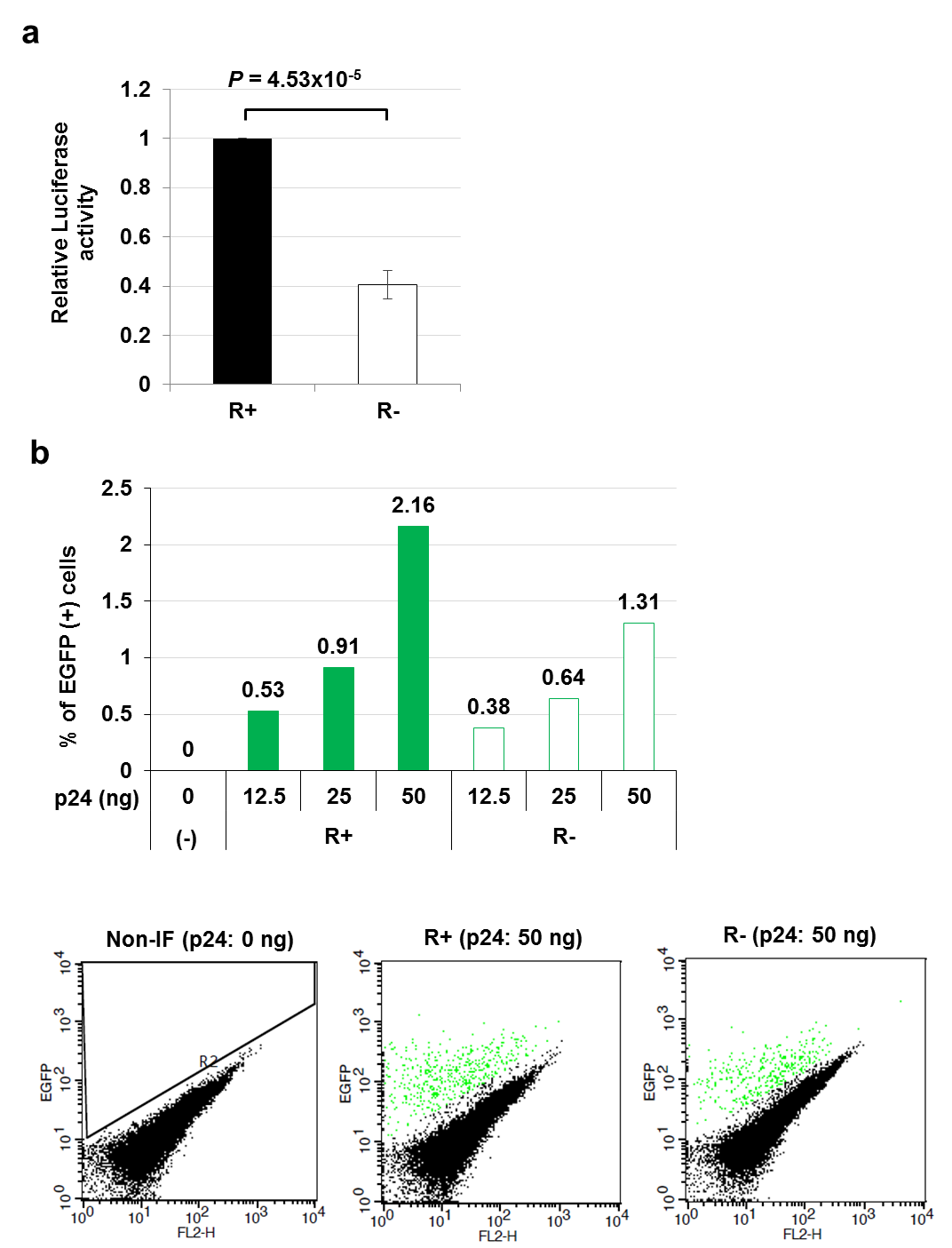

Supplement: Supplementary file 15 — Additional file 15: Figure S14. Vpr upregulates viral infection in differentiated MM-6 cells. a MM-6 cells were infected with Vpr proficient (R+) or deficient (R−) NL4-3-Luc/E- viruses. Luciferase assay was performed at 3dpi. Data were obtained from more than three independent experiments. Error bar indicates ± SEM. b MM-6 cells were infected with Vpr proficient (R+) or deficient (R−) NL4-3-EGFP/E- viruses. Percentage of EGFP positive cells was determined by FCM at 3dpi. Representative FCM data are shown in bottom panels. Green colored plots were gated as EGFP positive cells. [file 12977_2018_391_MOESM15_ESM.tif]

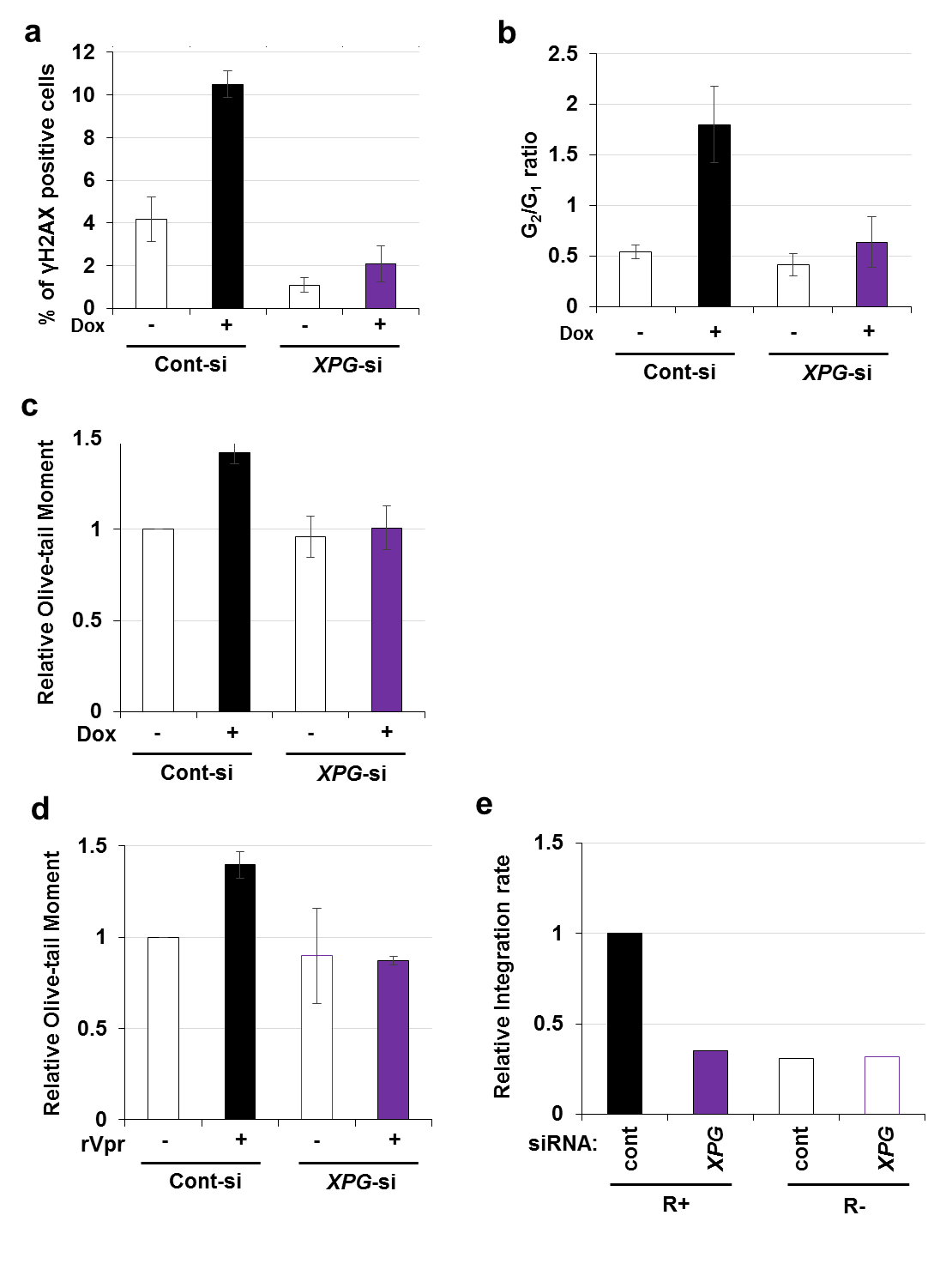

Supplement: Supplementary file 16 — Additional file 16: Figure S15. XPG is required for Vpr-induced DDR and DSB. a, b Effects of downregulation of XPG on Vpr-induced DDR. The expression of XPG in Mit-23 cells was first downregulated by siRNA, and then Vpr expression was initiated on day 2 after introduction of XPG targeting siRNA. On day 1 after Vpr expression was started, phosphorylation of H2AX (a) and G2/M checkpoint activation (b) was analysed by flow cytometry. Downregulation of XPG significantly reduced Vpr-induced phosphorylation of H2AX (P = 4.4 × 10^−5^), and the G2/M arrest (P = 0.025). Data were obtained from three independent experiments. Error bar indicates ± SEM. c XPG is required for Vpr-induced DSBs. Neutral comet assay was performed using Mit-23 cell, the XPG expression of which was down-regulated. Data were obtained from three independent experiments. Error bar indicates ± SEM. P = 0.037. d XPG is required for rVpr-induced DSB in resting macrophages. Differentiated MM-6 cells were treated with 100 ng/ml of rVpr under the down-regulation of XPG, and subjected to neutral comet assay. Data were obtained from three independent experiments. Error bar indicates ± SEM. P = 0.001. e XPG is required for Vpr-induced upregulation of viral infection in resting macrophages. Differentiated MM-6 cells were infected with Vpr proficient (R+) or deficient (R−) NL4-3 viruses after transfection of siRNA, and the integration rate was quantitated by Alu-gag two-step nested qPCR; relative integration rates are shown. A representative result out of two independent experiments is depicted. [file 12977_2018_391_MOESM16_ESM.tif]

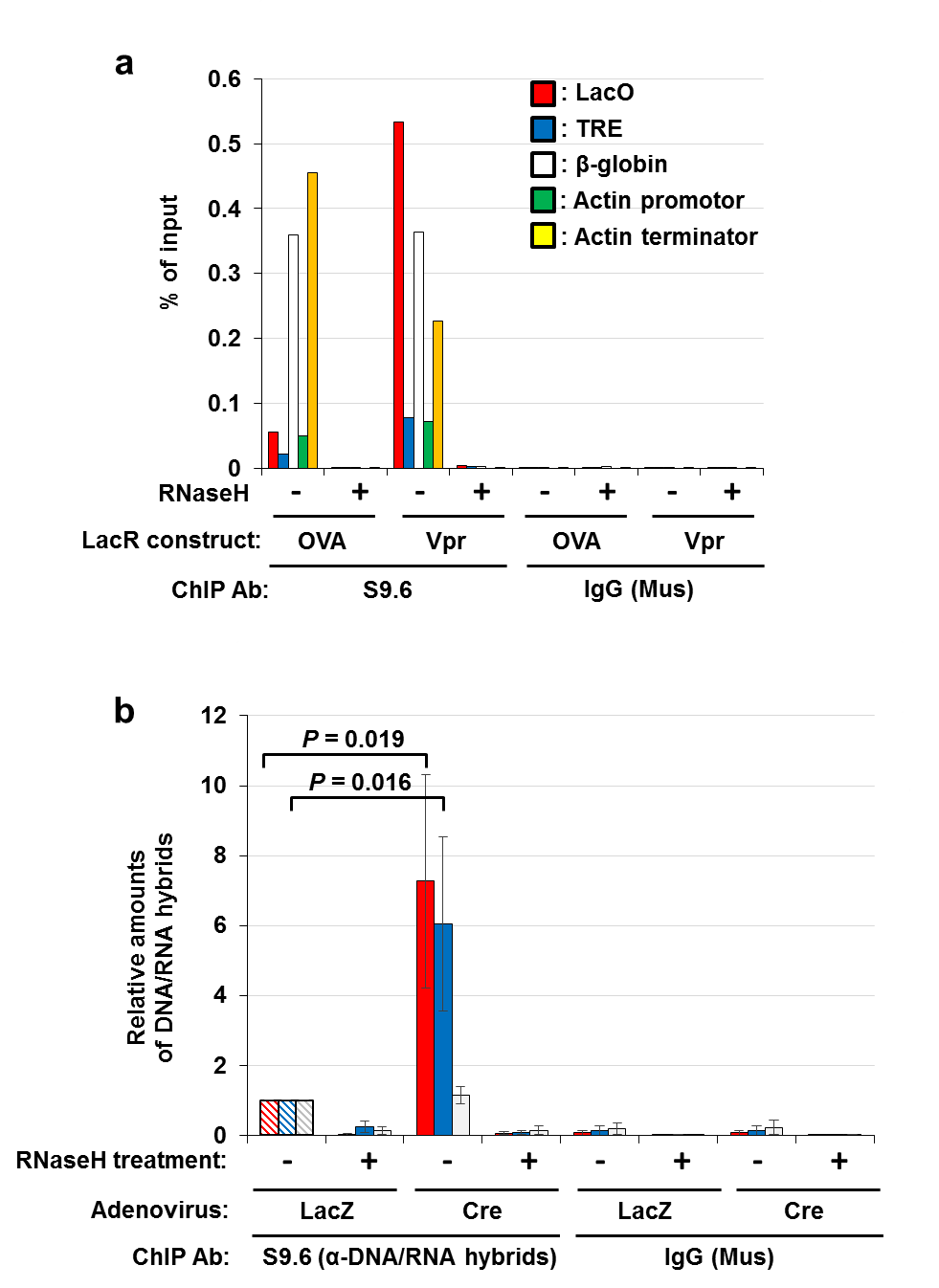

Supplement: Supplementary file 17 — Additional file 17: Figure S16. Forced accumulation of Vpr increases formation of R-loops. a U2OS/2-6-3 cells were transfected with indicated LacR fused constructs. Two days after transfection, DNA samples were prepared and subjected to DRIP assay. DRIP assay was done with α-DNA/RNA hybrids antibody (S9.6). RNaseH treatments were performed to confirm the specificity of DRIP assay. Analysis of promotor (green column) and terminator (yellow column) regions of Actin gene were included in the qPCR analysis, as negative and positive control of DRIP assay, respectively. A representative result out of two independent experiments is depicted. b 263/loxP-CLV cells were infected with Ad-LacZ or -Cre. DNA samples were prepared and subjected to DRIP assay at 2dpi. Data were obtained from more than three independent experiments. Error bar indicates ± SEM. [file 12977_2018_391_MOESM17_ESM.tif]

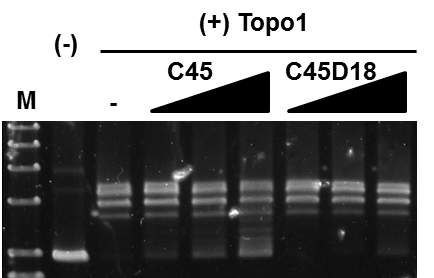

Supplement: Supplementary file 18 — Additional file 18: Figure S17. C-terminus of Vpr is required for topoisomer induction activity. DNA supercoiling assay was performed with increasing amounts of peptides (1, 10, 100 pmol; 0.015, 0.15, 1.5 Vpr molecules/bp). In the presence of C45, faster migrating topoisomers appeared. [file 12977_2018_391_MOESM18_ESM.tif]
